# Genetic Diversity and Genomic Plasticity of *Cryptococcus neoformans* AD Hybrid Strains

**DOI:** 10.1534/g3.111.001255

**Published:** 2012-01-01

**Authors:** Wenjun Li, Anna Floyd Averette, Marie Desnos-Ollivier, Min Ni, Françoise Dromer, Joseph Heitman

**Affiliations:** * Department of Molecular Genetics and Microbiology, Duke University Medical Center, Durham, North Carolina 27710; † Institut Pasteur, Unité de Mycologie Moléculaire, Paris, France; ‡ CNRS URA3012, Paris, France

## Abstract

Natural hybridization between two strains, varieties, or species is a common phenomenon in both plants and animals. Although hybridization may skew established gene pools, it generates population diversity efficiently and sometimes results in the emergence of newly adapted genotypes. *Cryptococcus neoformans*, which causes the most frequent opportunistic fungal infection in immunocompromised hosts, has three serotypes: A, D, and AD. Serotype-specific multilocus sequence typing and serotype-specific comparative genome hybridization were applied to investigate the genetic variability and genomic organization of *C. neoformans* serotype AD isolates. We confirm that *C. neoformans* serotype AD isolates are hybrids of serotype A and D strains. Compared with haploid strains, most AD hybrid isolates exhibit unique multilocus sequence typing genotypes, suggesting that multiple independent hybridization events punctuated the origin and evolutionary trajectory of AD hybrids. The *MAT***a** alleles from both haploid and AD hybrid isolates group closely to form a cluster or subcluster in both the serotype A and D populations. The rare and unique distribution of *MAT***a** alleles may restrict sexual reproduction between isolates of opposite mating types. The genetic diversity of the serotype D population, including haploid strains and serotype D genomes of the AD hybrid, is significantly greater than that of serotype A, and there are signatures of recombination within the serotype D population. Given that *MAT***a** isolates are relatively rare, both opposite-sex and same-sex mating may contribute to genetic recombination of serotype D in nature. Extensive chromosome loss was observed in AD hybrid isolates, which results in loss of heterozygosity in the otherwise-heterozygous AD hybrid genome. Most AD hybrid isolates exhibit hybrid vigor and are resistant to the antifungal drug FK506. In addition, the *C. neoformans* AD hybrid genome is highly dynamic, with continuous chromosome loss, which may be a facile route for pathogen evolution through which genotypic and phenotypic variation is generated.

Hybridization occurs in almost all sexually reproducing groups of organisms, and natural hybridization is a relatively common phenomenon in both plants and animals (Arnold 1997; Mallet 2005). At least 25% of plant species and 10% of animal species, mostly the youngest species, are involved in hybridization and potential introgression with other species (Mallet 2005). In plants, hybridization is an important evolutionary force that generates population diversity and drives speciation (Soltis and Soltis 2009); hybridization followed by chromosome doubling is one of the most common mechanisms of speciation in Angiosperms (Paun *et al.* 2009). Although most hybrids are disadvantaged as a result of genetic incompatibility, the surviving offspring may have a beneficial combination of their parental genotypes, allowing them to adapt to changing environments. This phenomenon, referred to hybrid vigor, has been well documented in sunflower species, *Dacus tryoni* (a tephritid fly), and stickleback fish (Lewontin and Birch 1966; Lucek *et al.* 2010; Rieseberg *et al.* 2003, 2007). The liger, a hybrid between a male lion (*Panthera leo*) and a tigress (*Panthera tigris*), exhibits prominent hybrid vigor in the animal kingdom. Reports of natural hybridization in fungi were rare until recently (Olson and Stenlid 2002). Interspecific hybrids allow the colonization of novel habitats and expanded host range in several plant fungal pathogens (Brasier *et al.* 1999; Hamilton *et al.* 2009; Inderbitzin *et al.* 2011; Moon *et al.* 2004) and contribute significantly to the generation of novel species and stress adaptability in the *Saccharomyces sensu stricto* complex (de Barros Lopes *et al.* 2002; Querol *et al.* 2003; Sipiczki 2008).

*Cryptococcus neoformans* is a ubiquitous fungal pathogen that causes meningoencephalitis and pneumonia in immunocompromised hosts. It is one of the most common causes of death in HIV-infected patients (Mitchell and Perfect 1995; Perfect and Casadevall 2002). On the basis of antibody-promoted slide agglutination assays of polysaccharide capsules, *C. neoformans* has been classified into three serotypes: serotype A, D, and AD (Dromer *et al.* 1993; Ikeda *et al.* 1996; Kabasawa *et al.* 1991). Serotype A is distributed globally and causes >95% of human infections (Mitchell and Perfect 1995), whereas serotype D is mostly found in Europe but has a sporadic global distribution (Dromer *et al.* 2007). Serotype AD isolates that react with both serotype A and serotype D antisera to capsular polysaccharides are hybrids of serotype A and D strains. Although AD hybrid isolates are generally regarded as less frequent causes of human infections (Kabasawa *et al.* 1991; Mitchell and Perfect 1995), a recent survey in Europe revealed that 19% of human infections are caused by AD hybrid strains (Viviani *et al.* 2006). The finding of an unexpectedly high prevalence of serotype AD infections may be explained through the application of polymerase chain reaction (PCR) assays in molecular detection of *C. neoformans* and is likely caused by the impact of hybridization on virulence potential. Interspecific hybrids of *C. neoformans* and *Cryptococcus gattii* have recently been identified in human infections and may be much more common than previously appreciated (Bovers *et al.* 2006, 2008; Viviani *et al.* 2006).

Most AD hybrid strains are diploid or aneuploid (Cogliati *et al.* 2006; Lengeler *et al.* 2001; Xu *et al.* 2002; Xu and Mitchell 2003). Molecular analyses provide evidence that AD hybrid strains are the result of the hybridization of serotype A and D strains, a phenomenon in which mating plays a central role (Xu *et al.* 2000, 2002; Xu and Mitchell 2003). *C. neoformans* has a bipolar mating system that is controlled by the mating type locus (*MAT*) (Frasier *et al.* 2004; Lengeler *et al.* 2002). Two opposite mating types, **a** and α, are known in *C. neoformans*, but *MAT*α predominates in clinical and environmental populations (Kwon-Chung and Bennett 1978). Mating has been observed between strains of opposite mating types in the laboratory. A filamentous dikaryon is produced after cell−cell fusion, and nuclear fusion occurs later in the fruiting body (the basidium), resulting in a transient **a**/α diploid state that immediately undergoes meiosis and sporulation to produce α and **a** haploid progeny (Kwon-Chung 1975, 1976). Although most serotype A isolates are exclusively *MAT*α, serotype A *MAT***a** strains have been recently identified in Botswana (Litvintseva *et al.* 2003, 2006), and there is evidence of sexual reproduction and genetic recombination in this population (Litvintseva *et al.* 2003). *C. neoformans* can also undergo unisexual mating, especially between α cells, to produce stable α/α diploids as well as haploid progeny (Lin *et al.* 2005, 2007).

It has been estimated that serotype A and D of *C. neoformans* diverged ∼18 million years ago and have ∼10% to 15% nucleotide polymorphism at the whole genome level (Kavanaugh *et al.* 2006; Sun and Xu 2009; Xu *et al.* 2002). In addition, genomic rearrangements make the genetic distance between these two serotypes even greater (supporting information, Figure S1) (Kavanaugh *et al.* 2006; Sun and Xu 2009). When serotype A and D strains are mixed under appropriate conditions in the laboratory, cell−cell fusion proceeds normally but meiosis is impaired because of their highly divergent genetic backgrounds (Lengeler *et al.* 2001; Sun and Xu 2007; Xu *et al.* 2000, 2002], thus resulting in few viable haploid progeny (Lengeler *et al.* 2001; Sun and Xu 2007). Despite impaired meiosis, AD hybrid cells resulting from these intravarietal matings can propagate mitotically as uninucleate diploid yeast cells after nuclear fusion. Thus, many natural AD hybrids remain in the diploid (or aneuploid) state (Lengeler *et al.* 2001). The authors of a previous study reported approximately 5% viability of the meiotic progeny of AD hybrids (Lengeler *et al.* 2001). The hybrids may have recombinant chromosomes rather than a simple combination of genomes from the two serotypes. The resulting recombinant genome may generate novel phenotypic traits. The laboratory-constructed AD hybrid strain exhibits greater virulence than parental serotype A and D strains *in vivo* and *in vitro*; they were resistant to ultraviolet light and have larger capsules (Lin *et al.* 2007). In some mouse infection experiments, *C. neoformans* AD hybrid strains exhibited intermediate or greater virulence than haploid serotype A and D (Barchiesi *et al.* 2005; Chaturvedi *et al.* 2002).

Genetic diversity and genomic organization may ultimately explain phenotypic variability of the pathogen, including, for example, host specificity, pathogenicity, antimicrobial resistance, and virulence. The genome organization of *C. neoformans* AD hybrids is largely unknown; however, the authors of a previous study found that some AD hybrid isolates are aneuploid on the basis of fluorescence-activated cell sorting (FACS) analysis, whereas others exhibit roughly the same DNA content as diploid control strains (Lengeler *et al.* 2001). Comparative gene genealogical analyses have provided evidence that the AD hybrid results from hybridization of serotype A and D strains (Xu *et al.* 2000, 2002, 2003). However, these studies were based on only two genes, *LAC1* and *URA5*, and a limited number of AD hybrid isolates were studied. The limited genetic information from these two genes may not be sufficient to investigate the origin and evolutionary trajectory of the AD hybrid. The limited number of available AD hybrid isolates may exclude some important evolutionary lineages that have yet to be isolated. In addition, whether hybridization between the two *C. neoformans* serotypes contributes to speciation is debatable (Soltis and Soltis 2009). Whole-genome duplication has been described in the *Saccharomyces* complex and it contributed greatly to speciation of this group by generating a number of novel species (Querol *et al.* 2003; Sipiczki 2008).

In this study, we explored the origin, population dynamics, evolutionary trajectory, and genomic plasticity of the *C. neoformans* AD hybrid by characterizing a large number of isolates in terms of genetic diversity and genomic organization using mating type- and serotype-specific PCR, multilocus sequence typing (MLST), and comparative genome hybridization (CGH). Our results reveal that serotype AD strains of *C. neoformans* result from multiple, independent hybridization events that yielded a highly dynamic hybrid genome. Thus, the AD hybrid is an important source of genotypic and phenotypic variation in *C. neoformans*.

## Materials and Methods

### Strains and media

In total, 64 *C. neoformans* isolates, including 31 serotype AD and 33 serotype D isolates (32 haploid and one diploid), are included in this study (Table 1). All strains were cultured on yeast extract-peptone-dextrose (YPD) agar medium at 30°. To conduct comparative phylogenetic analyses, DNA sequences of five MLST markers (*IGS*, *URE1*, *GPD1*, *LAC1*, and *MPD1*) in eight serotype A strains (2 VNI, 2 VNII, and 4 VNB) from previous studies were downloaded from GenBank.

**Table 1:** The mating type, serotype, and origin of *C. neoformans* isolates included in this study

Isolate	Mating type*^a^*	Serotype*^a^*	Origin
713	αADα	AD	Italy
42-10	αADα	AD	NC, USA
5-19	αADα	AD	NC, USA
6-20	αADα	AD	NC, USA
ATCC48184	**a**ADα	AD	Japan
CDC228	**a**ADα	AD	USA
CDC304	**a**ADα	AD	USA
CDC92-74	-ADα	AD	USA
CDC94-383	-ADα	AD	Unknown
CBS132	αAD**a**	AD	Italy
IT752	**a**ADα	AD	Italy
IT756	αAD**a**	AD	Italy
IUM92-4686	**a**AD-	AD	Italy
IUM92-6198	**a**ADα	AD	Italy
KW5	αAD**a**	AD	Kuwait
MMRL752	**a**ADα	AD	Italy
MMRL774	αAD**a**	AD	Italy
MMRL1351	-AD**a**	AD	France
MMRL1365	**a**ADα	AD	USA
NC34-21	**a**ADα	AD	NC, USA
ZG287	αAD**a**	AD	China
ZG290	**a**ADα	AD	China
AD6-93	**a**ADα	AD	Sub-Saharan Africa
AD7-97	**a**ADα	AD	North Africa
AD6-38	**a**ADα	AD	Europe/North Africa
AD7-91	-ADα	AD	Sub-Saharan Africa
AD7-85	-ADα	AD	Sub-Saharan Africa
AD7-69	αAD-	AD	Europe
AD7-75	αADα	AD	Europe
AD2-71	αADα	AD	Europe
AD7-95	αADα	AD	Europe
CDC92-27	Dα	D	USA
VANC.R461	Dα	D	Canada
431	Dα	D	Denmark
AD7-71	Dα	D	Europe
AD3-14	Dα	D	Europe/Africa
AD3-13	Dα	D	Europe/Africa
AD2-62	Dα	D	Europe
AD2-95	Dα	D	Europe
AD2-96	Dα	D	Europe
MMRL1076	Dα	D	USA
2-14	Dα	D	NC, USA
3-15	Dα	D	NC, USA
3-28	Dα	D	NC, USA
NIH12	Dα	D	Denmark
MMRL751	Dα	D	Italy
528	Dα	D	Italy
529	Dα	D	Italy
709	Dα	D	Italy
MMRL760	Dα	D	Italy
434	Dα	D	Denmark
MMRL757	Dα	D	Italy
3311	Dα	D	NC, USA
B3179	Dα	D	USA
CDC92-18	Dα	D	USA
Y290-90	Dα	D	Canada
2-22	Dα	D	NC, USA
CAP672	Dα	D	Unknown
NIH264	D**a**	D	USA
NIH276	D**a**	D	USA
NIH430	D**a**	D	USA
NIH433	D**a**	D	USA
AD8-62	αDDα	D	Europe

NC, North Carolina.

a The mating type and molecular serotype were determined by mating type- and serotype-specific PCR amplification of the *STE20* gene (see Figure S2).

### Ploidy analysis by fluorescence flow cytometery (FACS)

FACS analysis was performed as described previously (Tanaka *et al.* 1996). In brief, yeast cells were harvested after growth on YPD medium for 48 hr, washed once in phosphate-buffered saline buffer, and fixed in 1 ml of 70% ethanol overnight at 4°. Fixed cells were then washed with 1 ml of NS buffer (10 mM Tris-HCl [pH 7.6]; 0.25 M sucrose; 1 mM EDTA [pH 8.0]; 1 mM MgCl_2_; 0.1 mM CaCl_2_; and 0.1 mM ZnCl_2_) and then stained with propidium iodide (12 mg/ml) in 0.2 ml of NS buffer containing RNaseA (1 mg/ml) at 4° for 8 hr in the dark. Then, 0.05 ml of the stained cell preparation was diluted into 1.95 ml of 50 mM Tris-HCl (pH 8.0) and sonicated for 1 min. FACS was performed on 10,000 cells and analyzed on the FL1 channel of a Becton-Dickinson FACScan. Haploid isolate H99 and diploid isolate XL143 were included as controls.

### Genomic DNA isolation, PCR amplification, and DNA sequencing

After 2 days of growth on YPD agar, *C. neoformans* strains were collected directly for genomic DNA isolation. Genomic DNA isolation was performed using the MasterPure Yeast DNA Purification Kit (Epicentre Biotechnologies) with minor modifications. In brief, 500 μl of glass beads (425−600 nm) and 300 μl of cell lysis solution were added to the cells to assist cell wall disruption. Others steps followed the protocol provided by the manufacturer.

All PCR assays were conducted in a PTC-200 automated thermal cycler (MJ Research, Waltham, MA); 300 ng of DNA was amplified in a 25-μl reaction mixture containing 10 pM of each primer, 2 mM of each nucleotide (dATP, dCTP, dGTP, and dTTP), 2.5 μl of 10x Ex Taq buffer, 0.125 μl of Ex *Taq* polymerase (Takara, Shiga, Japan), and an appropriate volume of distilled water. The following conditions were used for the amplification: an initial 2 min of denaturation at 98°, followed by 35 cycles of denaturation for 10 sec at 98°, an annealing time of 15 sec at 54°, and an extension cycle for 1 min at 72°. The amplification was completed with an extension period of 5 min at 72°. Sterile water served as a negative control in each assay. PCR products were analyzed on 1% agarose gels (MLST and serotype/mating type determination) or 2% agarose gels (chromosome loss analysis).

Amplicons were purified using the QIAquick PCR Purification Kit (QIAGEN) as recommended by the manufacturer. PCR products were sequenced in both directions using BigDye Terminator version 3.1 cycle sequencing ready reaction mix (Applied Biosystems). Sequencing products were resolved using an ABI 3130 automated sequencer (Applied Biosystems). Sequences were assembled using Sequencher 4.8 (Gene Codes).

### Mating type and serotype identification

We initially determined the mating type and serotypes by PCR analysis of the *STE20*, *SXI1*α, *SXI2***a**, *PAK1*, *GPA1*, and *CNA1* genes as described previously (Lengeler *et al.* 2001). However, some AD hybrid strains are untypable because of PCR failure, which may be attributable to DNA sequence variation in the primer complementary regions. *STE20* is not only serotype-specific but also mating-type specific (Frasier *et al.* 2004), which makes it an appropriate marker for the determination of serotype and mating type of *C. neoformans*. New *STE20* primers were designed on the basis of DNA sequence alignment of *STE20* gene from strains JEC21 (Dα), JEC20 (D**a**), H99 (Aα), and 125.91 (A**a**; Table 2). Genomic DNA from strains JEC21, JEC20, H99, and KN99**a** served as positive controls, and sterile water was used as negative control. The mating type of most AD hybrid isolates was confirmed through mating assays on V8 and MS media (see the section *Mating assays*). Even with newly designed primers, the mating types of some AD hybrid isolates were still undeterminable because of negative PCR results. CGH analysis (see *RESULTS)* demonstrates that loss of the chromosome on which the *MAT* locus is located in AD hybrids results in negative PCR amplification of *STE20*. Thus, the use of a single marker, such as *STE20*, can in some cases misclassify AD hybrid isolates as haploid strains (without FACS data) or diploid isolates of a single serotype (combined with FACS analysis).

**Table 2:** Primers used in this study

Primer sequence, 5′-3′	Target gene	Chr, (D)/(A)	Amplicon size, bp	Purpose
GGAAAGGCTTAGGTAGTCTCCTTC	*IGS* (A)	2 (A)	1092	PCR and MLST sequencing
ATCCATGACCCCCAGGTTCACGAT				
GGAAAGGCTTAGGTAGTCTCCTTC	*IGS* (D)	2 (D)	1076	PCR and MLST sequencing
TGGAAACAGATCCATGATTTCAGA				
TCGACGAGAATTTAAGGGAAACTA	*URE1* (A)	14 (A)	1093	PCR and MLST sequencing
TCTACAGTCGAGTAGTTATTATCAC				
CGACGAGAATTTAAGGGAGACTAA	*URE1* (D)	8 (D)	1094	PCR and MLST sequencing
CCTACGATTCGAGTAGCTATTGTCAG				
TCCAGTCTTTCACTTTTTCCCACG	*GPD1* (A)	7 (A)	889	PCR and MLST sequencing
GTCATAGAATTGAGTGGCGGTAAT				
CTGTCGCTTGCGTCATCGTAGTAG	*GPD1* (D)	6 (D)	897	PCR and MLST sequencing
GTAAAATCGCAACGTACATAGGAG				
ATATCACGTGAGTCAGAATCGAAA	*LAC1* (A)	8 (A)	779	PCR and MLST sequencing
GTCAGTATCGGACTATTAATCTCC				
TCGCCAACCGCGGTTTCTGATAAC	*LAC1* (D)	7 (D)	901	PCR and MLST sequencing
GGGCTATCAACCCTTAAACTTTTT				
AATAACCCAGATCCCCAGACTAAG	*MPD1* (A)	8 (A)	1162	PCR and MLST sequencing
CCACAATATCCTTGGAAGTCTTAAA				
TCACTATCATCAGCGGTACCTTAC	*MPD1* (D)	7 (D)	1190	PCR and MLST sequencing
GCCACAATATCCTTGGAAGTTTTA				
CTAACTCTACTACACCTCACGGCA	*STE20* (A**a**)	5 (A)	457	Mating type/serotype determination
CGCACTGCAAAATAGATAAGTCTG				
GGCTGCAATCACAGCACCTTAC	*STE20* (Aα)	5 (A)	330	Mating type/serotype determination
CTTCATGACATCACTCCCCTAT				
CACATCTCAGATGCCATTTTACCA	*STE20* (D**a**)	4 (D)	728	Mating type/serotype determination
AGCTCTAAGTCATATGGGTTATAT				
CTTAATTCACAGCACCAGCCTA	*STE20* (Dα)	4 (D)	592	Mating type/serotype determination
GGTCATCACAGTCAGTCACCAC				
AGGCAGTCAACCTGCACTTACA	Intergenic region	7 (D)/8 (A)	526 (D)/472 (A)	Serotype determination
CTTCTATATTCGGGTCACAGCAC				
ACCTTCAAGACAGACACATGGTT	Intergenic region	12 (D)/4 (A)	840 (D)/1128 (A)	Serotype determination
AAGAAGGGCAGTATCAGGGTTAC				
GAGGCAGAACGGTTATCTAAAGGA	Intergenic region	1 (D)/1 (A)	227 (D)/227 (A)	Quntitative real-time PCR
CCAACTCTTCTCGTTGAGACTGAT				
GATAACAGTGGACCTTGCAATGAG	Intergenic region	4 (D)/1 (A)	277 (D)/277 (A)	Quntitative real-time PCR
TGACTCCCAGAGAGGATGATTATG				
AAGAAGCTCTCCGACAAGTTCATC	Intergenic region	9 (D)/1 (A)	343 (D)/343 (A)	Quntitative real-time PCR
GGTTTCGGTAGACAGAAGAGAAGG				

The amplicon size is based on the DNA sequences of strains JEC21 (serotype D) and H99 (serotype A).

(A), serotype A; (A**a**), serotype A, *MAT***a**; Aα, serotype A, *MAT*α; (D), serotype D; (D**a**), serotype D, *MAT***a**; Dα, serotype D, *MAT*α; MLST, multilocus sequence typing; PCR, polymerase chain reaction.

To resolve this problem, we also designed primers from other chromosomes for serotype determination. These primers were chosen from regions that are conserved between the serotype A and D genomes and following PCR can generate bands of distinct size from serotype A and D strains. Thus, this PCR assay generated two distinct bands from the serotype A- and D-derived genome of the AD hybrid isolates. To design these primers, we compared the serotype A and D chromosomes by using BLASTn (Altschul *et al.* 1997) and aligned them by using the ACT tool (Carver *et al.* 2005).

### MLST and phylogenetic analysis

Previously, 12 housekeeping genes (*MPD1*, *TOP1*, *MP88*, *CAP59*, *URE1*, *PLB1*, *CAP10*, *GPD1*, *TEF1*, *SOD1*, *LAC1*, and *IGS1*) were included in MLST for population genetics studies of *C. neoformans* serotype A strains (Litvintseva *et al.* 2003, 2006, 2007). For *C. neoformans* serotype D, no MLST typing system has been developed, although the *LAC1* and *URA5* genes have been used to study the population structure of serotype AD strains with a TA cloning strategy (Xu *et al.* 2000, 2002, 2003). Because AD hybrid isolates have both the serotype A and D genomes in the yeast cells, the previous MLST primers for serotype A amplify both serotype A and D alleles; therefore, the mixed amplicons should be separated by TA cloning. To test a large number of AD hybrid isolates, we designed a serotype-specific MLST system to amplify a specific serotype A or D allele from the AD hybrid isolates.

Taking advantage of 10% to 15% sequence diversity between serotype A and D, we chose primers in serotype-specific regions to amplify the MLST loci on the basis of DNA sequence alignments between strains H99 (Broad Institute) and JEC21 (GenBank accession no. NC_006670-NC_006683). The serotype-specific primers for the five genetic markers are listed in Table 2.

The sequence type is defined as a unique DNA sequence produced by the concatenation of five genetic markers. Here, each AD hybrid isolate is regarded as two composite genomes (serotype A and serotype D). DNA sequences were aligned by ClustalW (Thompson *et al.* 1994), and alignment was confirmed by MUSCLE (Edgar 2004) and manual inspection. The aligned and verified DNA sequences were then imported into Mega 3.0 (Kumar *et al.* 2004) and PhyML (Dereeper *et al.* 2008) to determine the phylogenetic organization of the aligned DNA sequences. The phylogenetic organization was edited in FigureTree (http://tree.bio.ed.ac.uk/software/figtree/).

### Mating assays

For mating assays, strains were pregrown on YPD medium for 2 days. The cells were then mixed on V8 or MS agar medium and incubated at 25° for several days. Hyphae and basidiospore formation was assessed by light microscopy every other day.

### Comparative genome hybridization

A *C. neoformans* array designed at Washington University in Saint Louis, Missouri, is widely used for gene expression analysis. However, given that the serotype A and D genomes share approximately 85% to 90% sequence identity, most array probes could hybridize with both genomes in the AD hybrid isolates. In addition, probes were designed on the basis of cDNA sequences for expression analysis. To select serotype-specific probes to study the genome organization of the AD hybrid isolates, we compared the probes from the serotype A and D genomes by using BLASTn (e value = 0.0001). Only the probes (70-mers) that exactly matched one serotype but diverged from the other serotype more than 35% were defined as serotype-specific probes. A total of 2140 and 1312 probes distributed over the 14 *Cryptococcus* chromosomes were selected as serotype A- and D-specific probes for CGH analysis, respectively.

Genomic DNA hybridization was performed as previously described (Alonso-Monge *et al.* 1999). Basically, genomic DNA was ultrasonicated to generate ∼500-bp fragments and purified with a DNA Clean and Concentrator kit (Zymo Research, CA). Then, 5 μg of DNA was used for Cy-3 dUTP or Cy-5 dUTP labeling reactions with the Random Primer/Reaction Buffer mix (BioPrime Array CGH Genomic Labeling System; Invitrogen, Carlsbad, CA). Labeled DNA from the sample (AD hybrid isolate) and the control (equal volume of H99 and JEC21 genomic DNA) was competitively hybridized to microarray slides containing 70 mer oligonucleotides for the *C. neoformans* JEC21 and H99 genomes (Lin *et al.* 2006). After hybridization, arrays were scanned with a GenePix 4000B scanner (Axon Instruments) and normalized using GenePix Pro V.4.0 and Genespring tools. The log2 ratio of R635/R532 value was plotted according to the H99 and JEC21 genome in Microsoft Excel.

### Pulsed-field gel electrophoresis of *C. neoformans*

Yeast cells from a purified single colony were grown overnight in 5 ml of YPD medium at 30°. Then, 100 ml of the culture were added to 50 ml of yeast nitrogen base minimal medium and grown to OD_600_ 0.5 at 30° in a shaking incubator. To suppress capsule formation, the medium was supplemented with 1 M NaCl. Yeast cells were pelleted, washed three times in 0.5 M NaCl/50 mM EDTA (pH = 8.0), resuspended in 9.5 ml of water with 0.5 ml of β-mercaptoethanol, and incubated for 1 hr at 37° with gentle mixing. Cells were pelleted again, resuspended in 4 ml of spheroplasting solution (1 M sorbitol/10 mM EDTA/100 mM sodium citrate), 100 µl of lysing enzyme solution (20 mg of Sigma-Aldrich [St. Louis, MO] lysing enzyme from *Trichoderma harzianum* in 100 µl of spheroplasting solution) was added, and cells were incubated for 2 hr at 37° with gentle mixing.

Complete spheroplasting was checked by mixing ∼10 ml of cell suspension with the same volume of 10% (vol/vol) SDS and gently pipetting to monitor the increase in viscosity that results from the release of high molecular weight DNA. Spheroplasts were pelleted at 1800 rpm for 10 min at 4°, washed twice with ice-cold spheroplasting solution, resuspended in the remaining spheroplasting liquid, and diluted to a concentration of 10^9^–10^10^ cells/ml. The spheroplasts were then mixed with three volumes of low-melting agarose (1% low melting agarose [Bio-Rad] in 0.125 M EDTA [pH 8.0]; 50°) and poured into molds. Agarose was solidified for 30 min at room temperature and for an additional hour at 4°.

Agarose plugs containing spheroplasts were removed from the molds and spheroplasts were lysed for 24 hr at 55° in lysing solution (0.5 M EDTA/10 mM Tris HCl [pH 10]/1% Sarcosyl). Plugs were washed three times for 15 min each with 0.5 M EDTA (pH 8.0) and stored in 0.5 M EDTA at 4°. A standard 1% gel (1% pulse field-certified agarose in 0.5 × 90 mM Tris HCl/64.6 mM boric acid/2.5 mM EDTA [pH 8.3]) was run in a Bio-Rad CHEF DRII apparatus. Plugs were placed into the wells of a standard 13 × 14 cm gel, sealed with low-melting agarose, and solidified for 30 min at 4°. The gel was run in 0.5 × 90 mM Tris HCl/64.6 mM boric acid/2.5 mM EDTA (pH 8.3) buffer at 12° with the following settings: initial A-time 75 s, final A-time 150 s, start ratio 1.0, run time 30 hr, mode to 10. initial B-time 150 s, final B-time 300 s, start ratio 1.0, run time 54 hr, mode to 11. The voltage was set to 125 V.

### Spot assay to determine sensitivity to antifungal drugs

Sensitivity to the antifungal drugs FK506 and fluconazole was assessed using a spot growth dilution assay. In brief, after growing in liquid YPD medium overnight at 30°, yeast cells were counted at OD_600_ and diluted with liquid YPD medium to the cell density OD_600_ = 0.5. Yeast cells were serially diluted and spotted on YPD plates with 1 µg/ml FK506 or 0.5 µg/ml fluconazole, respectively. After growth at 30° and 37° for 48 hr, the YPD plates with FK506 were examined for cell growth. The YPD plates with fluconazole were examined for cell growth after growth at 30° for 48 hr.

## Results

### Most *C. neoformans* AD hybrid isolates harbor the rare a mating type

**Figure 1:**
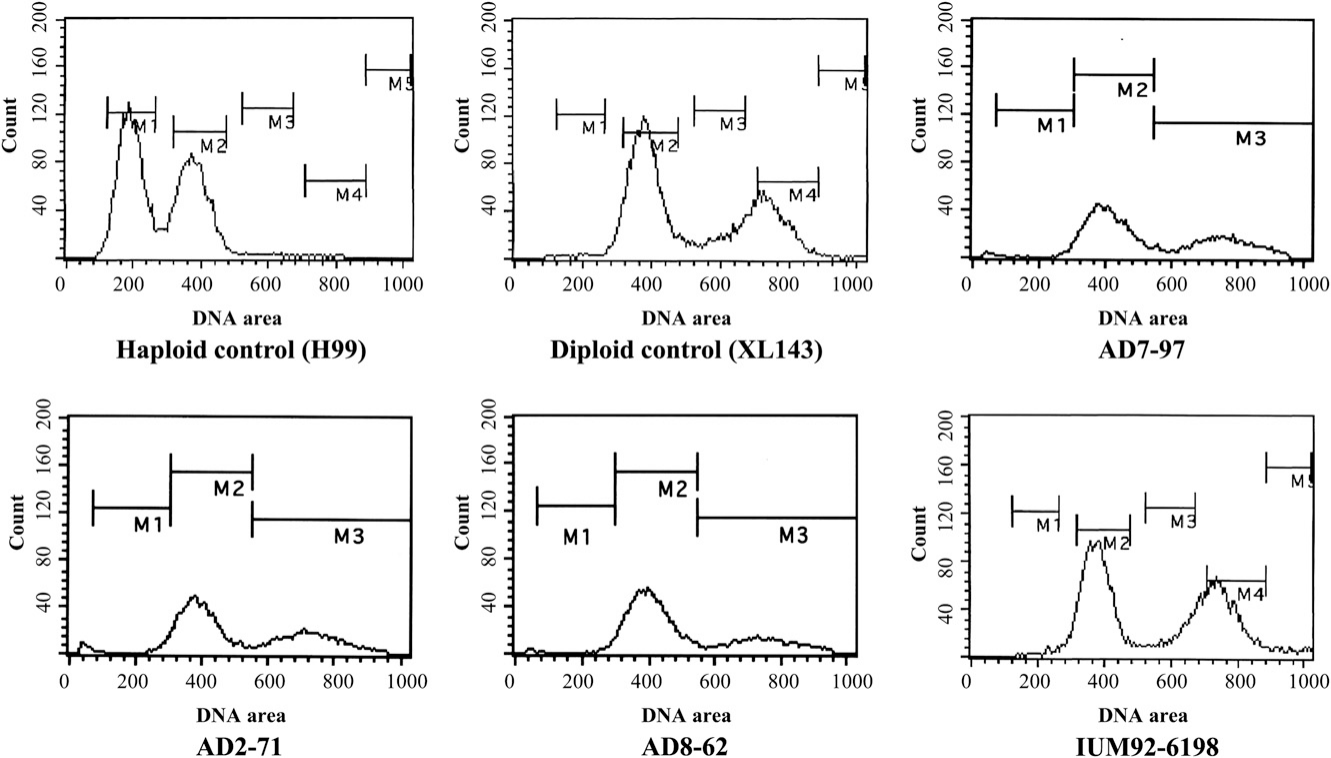
*C. neoformans* AD hybrid isolates have similar DNA content to diploid strains. DNA content was measured by FACS analysis. The haploid strain H99 and the diploid strain XL143 were used as 1N and 2N controls, respectively. The *C. neoformans* AD hybrid isolates AD7-97, AD2-71, and IUM92-6198 have similar DNA content as diploid control strain XL143 and serotype DD diploid strain AD8-62.

Of the 64 *C. neoformans* isolates included in this study, 32 exhibit a diploid profile, and 32 isolates were determined to be haploid based on FACS analysis (Figure 1). The genes in the *C. neoformans MAT* locus are classified into three categories: serotype-specific, mating type-specific, and serotype/mating type-specific genes (Frasier *et al.* 2004). The *STE20* gene that governs cell polarity at different stages of mating of *C. neoformans* is serotype/mating type-specific (Frasier *et al.* 2004; Nichols *et al.* 2004). Previously, *STE20* has been used for mating type and serotype determination (Lengeler *et al.* 2001; Lin *et al.* 2007); however, we were unable to PCR amplify the *STE20* gene from some AD hybrid isolates with existing primers. This may be partially explained by the sequence specificity of the *STE20* primers. We redesigned the *STE20* primers for serotype/mating type determination (Table 2). The mating type/serotype of 64 *C. neoformans* strains were identified by serotype- and mating type-specific PCR amplification of the *STE20* gene using these new primers (Table 1).

Of the 32 diploid isolates (on the basis of FACS analysis), 24 are AD hybrids as determined by evidence of amplification of the *STE20* genes from both serotypes (Figure 1, Table 1, and Figure S2). Among them, 12 are **a**ADα (39%), seven are αADα (23%), and five are αAD**a** (16%; Table 1). No **a**AD**a** isolates were identified. The *MAT***a** allele was identified in approximately 71% (17/24) of the AD hybrid isolates. Another eight isolates exhibited similar DNA content as diploid control strains in FACS but yielded only one serotype/mating type PCR product of the *STE20* gene, which suggests that these isolates are diploids of a single serotype due to genome duplication, or AD hybrids with either loss or divergence of the *STE20* gene. We also designed primers on other chromosomes to amplify PCR products of distinct sizes from each serotype. The primers chosen from conserved regions between serotype A and D chromosomes can amplify two fragments of distinct size from the AD hybrid isolates but only a single fragment from haploid or diploid mono serotype isolates. The PCR analysis allows the discrimination of AD hybrid isolates from both haploid and diploid isolates of single serotype. Together with the CGH analysis, we found that one of the eight isolates having similar DNA content as diploid isolates was a serotype DD diploid, whereas the other seven isolates were AD hybrid isolates that lost one copy of the chromosome on which the *MAT* locus is located (Chr 5 and Chr 4 in serotype A and D, respectively; see the section *Extensive chromosome loss in C. neoformans AD hybrid isolates*). Among the seven AD hybrid isolates, five lost the serotype A Chr 5 (4 “-ADα” and 1 “-AD**a**”), and two have lost the serotype D Chr 4 (one “**a**AD-” and one “αAD-”). The loss of one copy of the sex-related chromosome results in a failure to amplify the corresponding *STE20* gene.

Given that 17 of the 24 (71%) AD hybrid strains of known serotype/mating type in our study harbor the rare *MAT***a** allele (Table 1), our results suggest that AD hybrid isolates are a reservoir preserving the rare *MAT***a** mating type. Previously, other investigators found that serotype A *MAT***a** isolates are extant in Botswana (Litvintseva *et al.* 2007), but the *MAT***a** allele in the AD hybrid isolates has a broader distribution, including isolates from the United States and Europe (Table 1). Of note, at least three clinical isolates (**a**ADα) were recovered from patients born in or having traveled to Africa.

### Serotype A *MAT*a genome of AD hybrid isolates originates from Botswana

**Figure 2:**
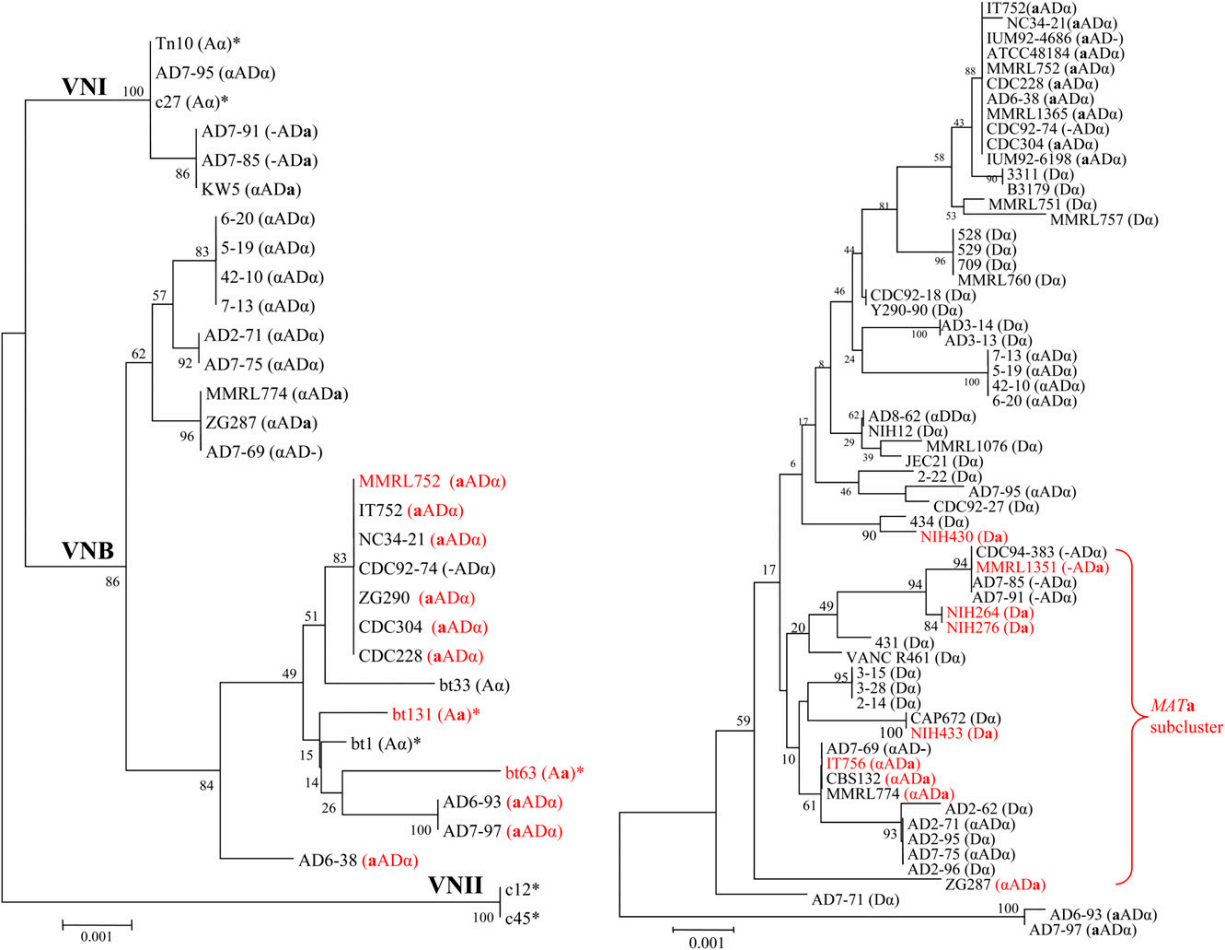
Phylogenetic organization of *C. neoformans* serotype A (left) and serotype D (right). This analysis was based on the concatenation of 5 MLST markers: *IGS*, *URE1*, *GPD1*, *LAC1*, and *MPD1*. The five loci were specifically amplified and sequenced from the serotype A and D genomes of the AD hybrid isolate by the use of serotype-specific primers. For comparison with haploid strains, 32 serotype D haploid strains, 1 serotype DD diploid strains, and 7 serotype A strains (*) of VNI, VNII, and VNB lineages from a previous study (Litvintseva *et al.* 2006) were included in the phylogenetic analysis. The concatenated DNA sequences were aligned by the use of ClustalW and MUSCLE. The phylogenetic organization was constructed with Mega 3.1 via the neighbor-joining method. The *MAT***a** strains are shown in red. The serotype and mating type of each isolate are indicated in brackets.

To analyze genetic variation of serotype A and D genomes of the AD hybrid isolates, we designed serotype-specific primers for MLST analysis by using DNA sequence comparisons between the JEC21 and H99 genomes (Table 2). To enable comparison with haploid serotype A and D strains, we also sequenced the five markers from 32 haploid serotype D strains and included five markers of serotype A strains from a previous study (Litvintseva *et al.* 2006). MLST identified two groups corresponding to the serotype A and D populations (Figure 2). Each group contained both haploid strains as well as AD hybrid strains, which suggests that AD hybrid isolates result from hybridization of serotype A and D strains.

*MAT*α predominates in *C. neoformans* natural populations, and Litvintseva *et al.* identified three serotype A lineages, VNI, VNII, and VNB (Litvintseva *et al.* 2006), with *MAT***a** isolates being strictly grouped in the VNB lineage (Litvintseva *et al.* 2003, 2006). Our MLST analysis based on five genetic markers grouped the serotype A *MAT***a** and *MAT*α-containing genomes into the VNB and VNI lineages, respectively (Figure 2). This strict distribution of the serotype A genome in the AD hybrid was not associated with the mating type of the serotype D genomic contribution. Our study further supports that the serotype A *MAT***a** genome of AD hybrid isolates originates from Botswana and that the serotype A *MAT*α genome possibly has a global distribution.

### Serotype D population has high recombination

In the MLST analysis based on five markers, we classified 62 serotype D genomes (including 29 AD hybrid isolates, 32 serotype D haploid isolates, and 1 serotype DD diploid isolate) into 31 STs (Table S1). In the MLST analysis we identified 40 STs among 123 serotype A genomes, including 100 from haploid strains in the previous study by Litvintseva *et al.* 2006 and 23 serotype A genomes from AD hybrid isolates in our study. The genetic diversity of the serotype D population is significantly greater than the serotype A population (*t*-test; *P* = 0.0139). The greater genetic diversity of serotype D is supported by low bootstrap values in the phylogenetic organization (Figure 2). The phylogenetic organization based on five markers of serotype D is distinct from each other (Figure S3). In addition, recombination is observed in the serotype D population based on the informative paired allele graphs of three MLST markers (*LAC1*, *GPD1*, and *MPD1*), showing that all four possible combinations are observed for alleles of any two genes (AB, ab, Ab, and aB; Figure S4). No recombination was observed in the serotype A population analyzed here.

To further characterize the possible roles recombination may play in the population structure of the serotype D population, we used MultiLocus software to examine the percentages of compatible loci and indices of association (I_A_). The analysis of both the percentage of compatible loci and I_A_ produce *P*-values that, if significant, reject the null hypothesis of recombination. If the *P*-values are not significant, the null hypothesis cannot be rejected and recombination can be inferred. When we performed the recombination test by using the MultiLocus software, the results suggested that random recombination of the serotype D population could not be rejected on the basis of the genotyping profile of three loci (*LAC1*, *GPD1*, and *MPD1*; I_A_ = 0.0531401 and *P* = 0.3). These results suggest that the serotype D population has a greater level of recombination than serotype A. Both opposite- and same-sex mating, which are observed in serotype D strains in the laboratory, may contribute to the population dynamics of serotype D isolates.

Although the serotype D population is highly diverse based on MLST, most serotype D *MAT***a** strains, including haploid strains and the serotype D genome from AD hybrid isolates, are grouped closely together to form a subcluster containing strains of both mating types (Figure 2). Recombination tests via the use of the MultiLocus software suggested that random recombination in this subgroup could not be rejected on the basis of the genotyping profile of three loci (*LAC1*, *GPD1*, and *MPD1*; IA = 0.00114586 and *P* = 0.398). This phylogenetic organization is similar to that of the serotype A *MAT***a** population and suggests that the serotype D *MAT***a** genome in AD hybrid isolates originate from serotype D haploid strains.

However, only four serotype D *MAT***a** isolates were included in the phylogenetic analysis, and the geographic origins of these isolates are unknown. This does not allow us to speculate about whether the serotype D *MAT***a** isolates are also limited to some regions. However, the serotype D genome of the AD hybrid isolates has a wide distribution including China, Italy, and Kuwait. In addition, two serotype D genomes from two AD hybrid isolates (AD6-93 and AD7-97: **a**ADα) distinctly form a unique cluster that was supported by a high bootstrap value (Figure 2). This unique lineage in the serotype D population may be a sign of an ancient hybridization event followed by several independent evolutionary paths of the AD hybrid. Alternatively, haploid isolates corresponding to this lineage may remain to be found or have become extinct since hybridization.

Although serotype A and D genomes of the AD hybrid isolates are phylogenetically grouped with serotype A and D haploid isolates, respectively, and exhibited comparable phylogenetic organization, most AD hybrid isolates have unique sequence types for both serotype A and D genomes in comparison to the haploid isolates. Only two AD hybrid isolates (AD7-75 and AD2-71) share the same serotype D ST (ST24) with two serotype D haploid isolates (AD2-95 and AD2-96; Table S1). This finding suggests that after the initial hybridization event, the AD hybrid isolates underwent independent evolution, which drives the AD hybrid isolates to become genetically diverse from their parental haploid isolates while still being phylogenetically related.

### Extensive chromosome loss in *C. neoformans* AD hybrid isolates

**Figure 3:**
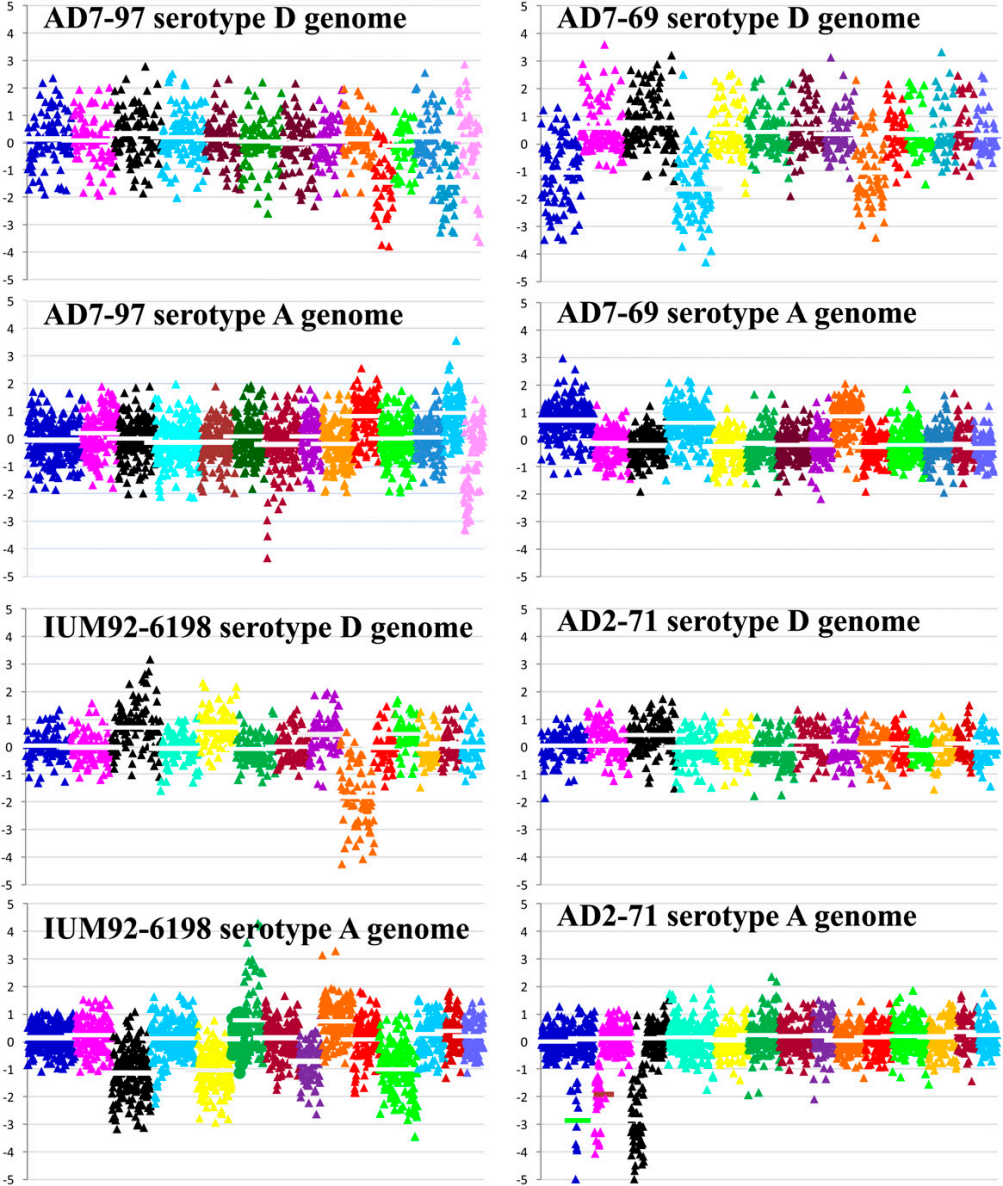
Extensive chromosome loss in *C. neoformans* AD hybrid isolates. Genomic DNA of the AD hybrid isolate and the control (equal amount of genomic DNA of H99 and JEC21) were competitively hybridized to 70-mer oligonucleotide probes designed from the JEC21 and H99 genomes. The serotype-specific probes on the array slide were determined on the basis of bioinformatic analysis of the probe sequences against the H99 and JEC21 genomic sequences (see Materials and methods section). The hybridization signal of serotype-specific probes was analyzed by the use of GenePix and GeneSpring software to produce the log2 ratio of R635/R532 (AD hybrid/control) value and plotted in Microsoft Excel. Each block of different color represents a chromosome. For the serotype D genome, from left to right the 14 blocks match chromosomes 1 to 14. Serotype A chromosome numbers are redefined according to the corresponding serotype D chromosome (for the corresponding serotype A and D chromosomes, see Figure S1). Each triangle represents a serotype-specific probe. The line in each block (chromosome) represents the average log2 ratio of genomic hybridization. Average log2 ratios lower and higher than 0.5 reflect chromosome loss and duplication, respectively. For example, strain IUM92-6198 lost serotype D Chr 9, and serotype A Chr 3, Chr5, Chr 8, and Chr 11. Strain AD2-71 has partial loss of serotype A Chr 1, Chr 2, and Chr 3.

Previous investigators found that AD hybrid isolates are monomorphic or homozygous at some loci but are heterozygous at many loci, including the *MAT* locus (Lengeler *et al.* 2001). However, information on the genomic organization of AD hybrids is limited. *C. neoformans* AD hybrid isolates are known to result from hybridization of serotype A and serotype D genomes; however, the genome organization of serotype AD strains is mainly unclear. Hu *et al.* (2008) used tiling array-based CGH to identify chromosome loss events in three AD hybrid isolates (two **a**ADα and one αAD**a**) that have opposite mating types of serotype A and D genomes. In our study, whole-genome hybridization analysis of the AD hybrid isolates demonstrated that *C. neoformans* AD hybrid isolates (**a**ADα, αAD**a**, and αADα) have extensive chromosome loss of either the serotype A or serotype D chromosomes (Figure 3). Both whole and partial chromosome loss is observed, which may generate novel chromosomes (Figure 3). Chromosome loss based on CGH analysis was confirmed by PCR (Figure S2). The PCR primers located in regions conserved between the serotype A and D genomes were used to amplify a polymorphic region with size variation between serotypes A and D, resulting in two different products of distinct sizes. Chromosome loss in AD hybrid isolates results in a single product in this PCR assay.

Our FACS results demonstrated that all of the AD hybrids have about the same DNA content as diploid isolates (Figure 1), although in CGH analysis we detected extensive chromosome loss (Figure 3). To explore this, CGH analysis found an approximately twofold log2 ratio (corresponding to copy number) of the chromosomes with loss of heterozygosity in comparison with the chromosomes that have both the serotype A and D parental chromosomal homologs (Figure 3), and thus the monomorphic chromosome has undergone duplication after the loss of one parental chromosome (Figure 3).

**Figure 4:**
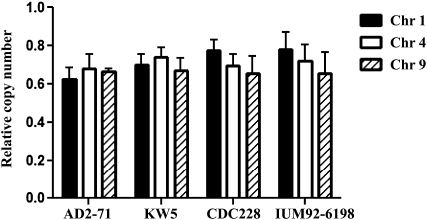
Quantitative analysis of gene copy number of AD hybrid isolates. Heterozygous loci of AD hybrid isolates have two copies of a gene, one from the serotype A parent and the other one from the serotype D parent. The primers used for quantitative analysis of the gene copy number of the AD hybrid isolates are located in regions conserved between serotype A and D and can amplify both serotype A and D loci. The primers target three loci on Chr 1, Chr 4, and Chr 9. On the basis of CGH, strain IUM92-6198 has lost serotype D Chr 9, and strains KW5 and CDC228 have lost serotype D Chr 1; however, these strains have an identical copy number of genes on Chr 1, Chr 4, and Chr 9. This finding indicates chromosome duplication of the remaining chromosomes after chromosome loss from one parental serotype in the AD hybrid.

To test whether the remaining chromosome was duplicated, we used quantitative real-time PCR to investigate the copy number of genes located on chromosomes that were lost. Our real-time PCR primers were selected from conserved regions of the serotype A and serotype D genomes and can amplify both serotype A and D alleles (~250 bp). We compared the copy number of genes that are homozygous (one serotype chromosome was lost) and those that are heterozygous. An identical gene copy number was obtained from heterozygous *vs.* homozygous genes (Figure 4), suggesting that duplication of the remaining chromosome follows chromosome loss from one serotype in the AD hybrid isolates. This further explains the greater log2 ratio of the remaining chromosome and the similar DNA content of the AD hybrid isolates as diploid strains based on FACS analysis.

### The genome of *C. neoformans* AD hybrid isolates is highly dynamic

**Figure 5:**
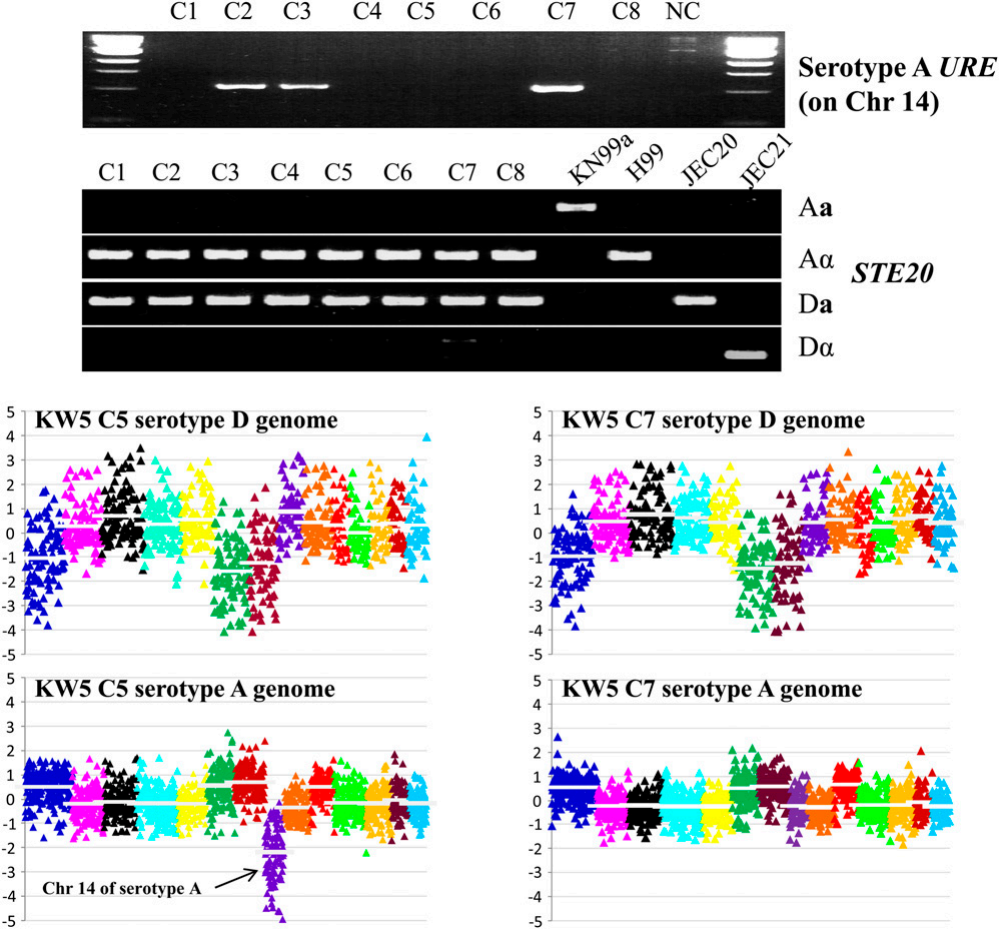
The serotype AD population exhibits genotypic plasticity (2N to 2N-1 to 2N). Two populations are derived from KW5: one with and one without serotype A Chr 14. The serotype A *URE1* gene, located on serotype A Chr 14, was amplified from three clones (C2, C3, and C7) from KW5 but not the other 5 clones. All eight clones have the same serotype/mating type, αAD**a**, based on serotype/mating type determination via the use of PCR assays of the *STE20* gene. CGH analysis demonstrates that clone C5 has lost serotype A Chr 14, which is extant in the C7 colony.

A previous study found that AD hybrid strain KW5 lost serotype D Chr 1, Chr 6, and Chr 7 and serotype A Chr 14 (Hu *et al.* 2008). However, an MLST marker from serotype A Chr 14 (*URE1*) was positive in isolate KW5 (Figure 5). We first considered that this might be a false-positive PCR because of DNA contamination. We repurified strain KW5 and isolated eight single colony subclones. Genomic DNA was isolated from these subclones for PCR, sequencing, and CGH analysis. PCR amplification of the serotype A *URE1* gene failed for clones C1, C4, C5, C6, and C8, whereas clones C2, C3, and C7 yielded a PCR product (Figure 5). Serotype/mating type specific PCR demonstrated that all eight clones shared the same mating type and serotype (αAD**a**; Figure 5). This led us to speculate that the KW5 strain may be a mixture of two groups of isolates, one group that has lost and another that has retained the serotype A Chr 14. However, this does not exclude possible contamination by another isolate. We therefore sequenced in all eight clones the most variable MLST marker, *IGS*, which is unique in strain KW5 compared with the other isolates in our strain collection. The *IGS* sequences of the eight clones are identical, unique, and the same as the original KW5 strain. Furthermore, CGH demonstrated that clone C5 lost the serotype A Chr 14 compared to clone C7 (Figure 5). They have highly similar chromosomal profiles for all other chromosomes. Based on these analyses, we conclude that the populations derived from isolate KW5 are a mixture of two groups of isolates, one with serotype A Chr 14 and another group that has lost serotype A Chr 14.

**Figure 6:**
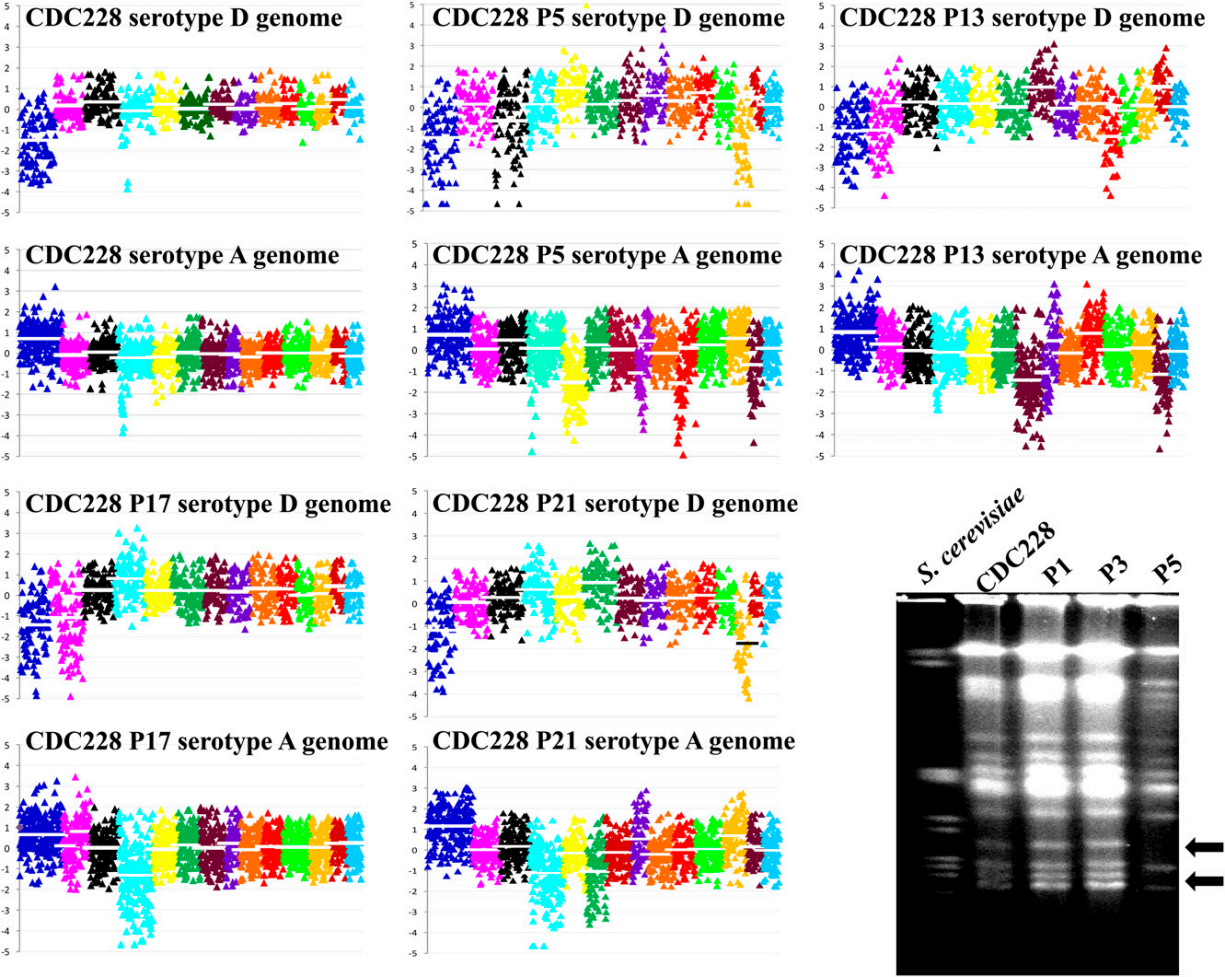
The progeny of self-fertile strain CDC228 (**a**ADα) have highly diverse genome organizations compared with the parental strain. Progeny P5, which is sensitive to the antifungal drug FK506 (Figure 7), has lost serotype D Chr 3 and Chr 12, and serotype A Chr 5, Chr 8 (partial), Chr 10 (partial), and Chr 13 compared with the parental strain CDC228, which only lost the serotype D copy of Chr 1 based on CGH. The P5 progeny has a distinct genomic profile based on CHEF analysis. It lacks some chromosomes (arrows) compared with CDC228.

**Figure 7:**
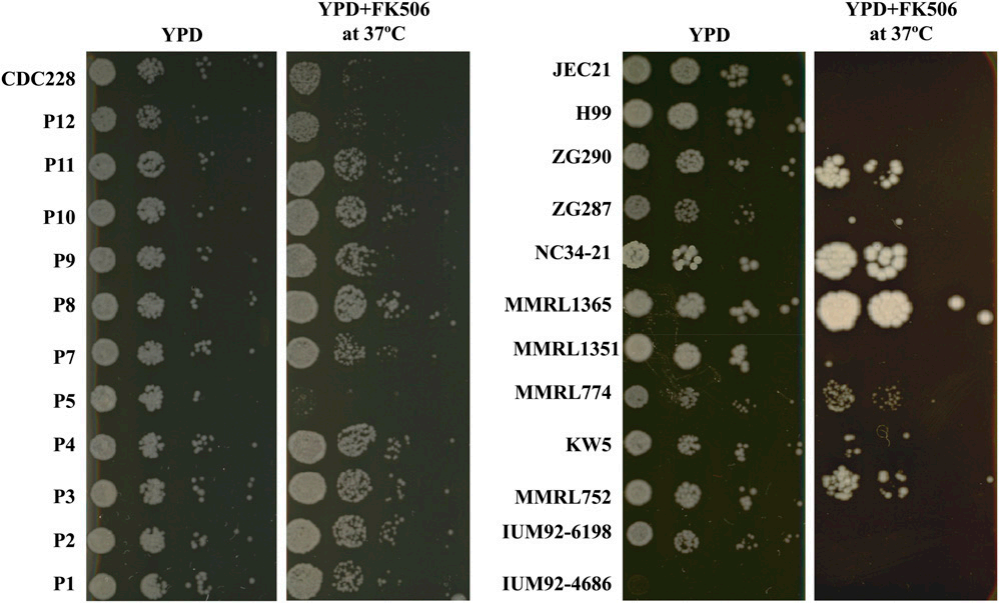
AD hybrid isolates exhibit phenotypic plasticity. Most AD hybrid isolates are resistant to the antifungal drug FK506 (left) and the progeny of the self-fertile strain CDC228 (**a**ADα), which is resistant the antifungal drug FK506, have altered FK506 resistance (right). After growing for 48 hr on YPD + FK506 (1 µg/ml) medium at 37°, five AD hybrid isolates (ZG290, NC34-21, MMRL1365, MMRL774, and MMRL752) exhibited resistance compared with the H99 and JEC21 haploid strains (left). After growing for 48 hr on YPD + FK506 (1 µg/ml) medium at 37°C, progeny P5 was sensitive to FK506 (right).

We further explored the genome dynamics of another AD hybrid isolate, CDC228 (**a**ADα), which is self-fertile on mating media (Lengeler *et al.* 2001). In a previous study, 21 CDC228 progeny obtained on V8 mating medium were dissected (Lengeler *et al.* 2001). Four CDC228 progeny were investigated here by CGH. Each progeny has a distinct genome organization (Figure 6). The parental strain CDC228 lost only serotype D Chr 1, but the progeny isolate P5 additionally lost serotype D Chr 3 and Chr 12 and serotype A Chr 5, Chr 8 (partial), Chr 10 (partial), and Chr 13 (Figure 6). The CGH results of strain CDC228 and its progeny, and KW5 and its derivatives, show that the AD hybrid genome is not stable and that genomic organization is dynamic and may contribute to phenotypic variation. We further tested the progeny of CDC228 and observed that some progeny lost the resistance to FK506 characteristic of CDC228 (Figure 7; as mentioned in the section *Many C. neoformans* *AD hybrid isolates are resistant to the antifungal drug FK506*).

### Many *C. neoformans* AD hybrid isolates are resistant to the antifungal drug FK506

We tested the susceptibility of *C. neoformans* AD hybrids to two antifungal drugs, fluconazole and FK506. Our results demonstrate that most AD hybrids are resistant to FK506 (Figure 7), although they remain sensitive to fluconazole (data not shown). The self-fertile AD hybrid isolate CDC228 is also resistant to FK506. Given that the CDC228 progeny have extensive chromosome loss and reorganization (Figure 6), the question is whether the genome reorganization contributes to phenotypes such as resistance to antifungal drugs. Spot dilution growth assays on FK506 medium demonstrated that some CDC228 progeny maintain whereas others lost the FK506 resistance phenotype (Figure 7). We further studied the chromosome organization of some progeny of distinct phenotype by separating chromosomes on a CHEF gel and observed that the CDC228 progeny P5, which lost the FK506 resistance phenotype, is missing some chromosomes compared with the parental strain and other progeny (Figure 6). This novel genomic organization may result in the loss of resistance to FK506.

## Discussion

Hybridization and its role in population dynamics and speciation have been widely investigated in plants and animals (Dowling and Secor 1997; Soltis and Soltis 2009). It has also been shown that intraspecific and interspecific hybridization of plant fungal pathogens leads to host expansion and adaptation to novel environmental niches (Hamilton *et al.* 2009; Moon *et al.* 2004; Olson and Stenlid 2002); in addition to sexual reproduction, this may be an important biological force driving evolution of plant fungal pathogens. In comparison, hybrids of human fungal pathogens are less characterized and understood. Both intervarietal (AD hybrid) and interspecies hybrids (*C. neoformans* x *C. gattii* hybrid) of *C. neoformans* have been either isolated from nature or patients or constructed in the laboratory (Bovers *et al.* 2006, 2008; Cogliati *et al.* 2006; Lengeler *et al.* 2001; Lin *et al.* 2007; Tanaka *et al.* 1996; Viviani *et al.* 2006; Whelan and Kwon-Chung 1986). A recent study in Europe revealed that approximately 19% of cryptococcosis cases are caused by AD hybrid isolates (Viviani *et al.* 2006). Given the high prevalence of *C. neoformans* hybrids in human infections, hybridization may play a fundamental role in the evolution of this fungal pathogen. Indeed, mixed infection of serotype A and D isolates as well as isolates of the same serotype but with different molecular types or mating types have been documented (Desnos-Ollivier *et al.* 2010; Mandal *et al.* 2005; Sullivan *et al.* 1996). The high rate of mixed infection may provide an opportunity for hybridization in the host in addition to routes to hybridization in nature.

Comparative gene genealogical analyses suggest that AD hybrids result from hybridization of serotype A and D strains (Xu *et al.* 2000, 2002, 2003). In our study, the serotype A and D genomes of the AD hybrid isolates group with haploid serotype A and D isolates, respectively, on the basis of phylogenetic analysis of five MLST markers (Figure 2). The consensus phylogenetic organization of haploid isolates and AD hybrid isolates confirms a recent hybridization origin of the AD hybrid. However, the serotype A and D genomes of most AD hybrid isolates are genotypically distinct from the haploid isolates, except for two AD hybrid isolates (AD7-75 and AD2-71) that share the same sequence type (ST24) with two serotype D haploid isolates (AD7-91 and AD7-85; Table S1). The consistent phylogenetic organization but genotypic divergence between the AD hybrid and haploid isolates supports the role of hybridization in the origin of the AD hybrid but also suggests ongoing independent evolutionary trajectories. In addition, the serotype D genomes of two AD hybrid isolates (AD6-93 and AD7-97: **a**ADα) exclusively form a basal lineage in the serotype D population (Figure 2). Although haploid serotype D isolates belonging to this lineage may remain to be found or be extinct, the AD hybrid isolates in this lineage may result from an ancient hybridization event followed by independent evolution to form a distinct lineage. However, the serotype A genomes of these two AD hybrids are classified into the VNB lineage and are closely related to isolate Bt63 (*MAT***a**) from Botswana.

On the basis of MLST, three lineages (VNI, VNII, and VNB) have been previously identified in the serotype A population (Litvintseva *et al.* 2006). The serotype A *MAT***a** and *MAT*α genomes of AD hybrid strains group into the VNB and VNI lineages, respectively (Figure 2). Given that the serotype A *MAT***a** VNB isolates are mainly identified in Botswana (Litvintseva *et al.* 2006), it is hypothesized that the serotype A *MAT***a** genome of the AD hybrid strains originates from Botswana, whereas the *MAT*α genome of the AD hybrid has a global distribution. Our result is in accord with a previous analysis of the serotype A genome of the AD hybrid isolates, in which three MLST markers (*CAP10*, *URE1*, and *GPD1*) were used (Litvintseva *et al.* 2007). Similarly, the *MAT***a** genomes from both the serotype D haploid isolates and AD hybrid isolates group closely to form a subcluster (Figure 2). The limited number of serotype D *MAT***a** isolates included in the phylogenetic analysis and the unknown geographic origin of the serotype D *MAT***a** haploid isolates (which are very likely from Europe where most serotype D isolates were identified) do not allow us to conclude that serotype D *MAT***a** genomes of the AD hybrid originate from a restricted geographic region as is the case with the serotype A *MAT***a** genome. Identification and analysis of more serotype D *MAT***a** isolates will be necessary to address this question in the future.

Given the strict distribution of serotype D *MAT***a** strains in the phylogenetic organization and the limited number of *MAT***a** strains in nature, the serotype D population is assumed to be relatively clonal except the subcluster containing both *MAT***a** and *MAT*α isolates. Indeed, random recombination in the subcluster containing both *MAT***a** and *MAT*α isolates could not be rejected on the basis of genotyping with three MLST markers (*LAC1*, *GPD1*, and *MPD1*; I_A_ = 0.00114586, and *P* = 0.398). This finding reveals ongoing recombination in this subcluster that may be attributable to sexual reproduction of strains of opposite mating types. However, our recombination test using MultiLocus software also suggests ongoing recombination in the haploid serotype D population on the basis of three MLST markers (*LAC1*, *GPD1*, and *MPD1*; I_A_ = 0.0531401, *P* = 0.3). In addition, the serotype D population (31 STs among 62 genomes) has a significantly greater genetic diversity than the serotype A population (40 STs among 123 genomes; *t*-test, *P* = 0.0139). High genetic diversity within a population is often associated with sexual reproduction in a sexually compatible microorganism (Campbell and Carter 2006). Although the serotype D population is dominated by *MAT*α isolates, sexual reproduction between *MAT*α isolates is observed in the laboratory (Lin *et al.* 2005). Same-sex mating may contribute to increase genetic diversity of the serotype D population in nature. Same-sex mating of serotype A strains has not been observed in the laboratory thus far, but population genetic evidence supports that it occurs in nature, but may be less frequent. If so, this could explain in part why the serotype D population is more diverse than serotype A.

Of the 31 AD hybrid isolates included in this study, 24 have an identified serotype/mating type. The serotype/mating type of the remaining seven AD hybrid isolates could not be determined because of the loss of the mating type chromosome of one serotype (Table 1). Of the 24 AD hybrid isolates with designated serotype/mating type, seven had only one mating type (*MAT*α; αADα) and 17 had both mating types (12 **a**ADα and 5 αAD**a**) present in their diploid genomes. αADα hybrid isolates originate from unisexual mating and hybridization of *MAT*α strains of serotype A and D strains (Lin *et al.* 2007). On the contrary, **a**ADα and αAD**a** hybrid isolates are products of opposite-sex mating and hybridization events. Thus, there have been at least three independent AD hybridization events inferred based on only the analysis of *MAT* configuration. No **a**AD**a** hybrid isolates have been identified in nature thus far, although they can be constructed in the laboratory (Lin *et al.* 2008). This may be explained by the rarity of *MAT***a** isolates in the population or that same-sex mating is less common between *MAT***a** isolates (Lin *et al.* 2008).

Previous authors found that AD hybrids are heterozygous at some loci but homozygous at others (Cogliati *et al.* 2006; Lengeler *et al.* 2001). On the basis of serotype-specific CGH, we provide strong evidence that the homozygous chromosomes in AD hybrid isolates result from the loss of the chromosome from the genome of one serotype (Figure 3). Most chromosomes, including the mating type chromosome, have the potential to be lost. However, chromosomes 2, 6, 7, and 8, on which the five MLST markers are located, seem to be more stable than others on the basis of our analyses. On the basis of the chromosomal synteny between the serotype A and D genomes (Figure S1), chromosomes 3 and 11 are less collinear because of a translocation. Chromosome 9 has a significant inversion between the two serotypes. In addition, Chr 1 seems to be more frequently lost from the serotype D genome, which results in homozygosity for the serotype A Chr 1 typically by reduplication (Figure 3). Disomy for Chr 1 in haploid strains (1N+1) has been recently associated with heteroresistance to fluconazole in *C. neoformans* serotype A (Sionov *et al.* 2010). However, we found that AD hybrid isolates are generally not more resistant to fluconazole than haploid isolates (data not shown).

Our results demonstrate that the genomic organization of the AD hybrid isolates is highly complex and diverse rather than a simple combination of genomes from the two parents, and this highly diverse genomic organization significantly increases the population diversity of *C. neoformans*. Moreover, we observed that the genomic organization of AD hybrid isolates is highly dynamic with great plasticity. Mitotic propagation of AD hybrids can generate population diversity, including the loss or duplication of whole and partial chromosomes as well as reorganization of the chromosomes. The AD hybrid isolate KW5 is an example demonstrating the complexity of genome dynamics in *C. neoformans* AD hybrids. Our study demonstrated that populations derived from the KW5 isolate are a mixture of at least two groups, one with and the other without the serotype A copy of Chr 14 (Figure 5). When and how the serotype A Chr 14 was lost from the subpopulation is not known. Isolate CDC228 (**a**ADα) is another example of genomic diversity in an AD hybrid. This strain is self-fertile, and no mating partner is required in this process. Although the viability of the meiotic progeny is low, the surviving progeny are highly diverse in genomic organization (Figure 6). This is an efficient route for *C. neoformans* to generate diversity, which may contribute to the ability to adapt to changing environments. The parental AD hybrid isolate CDC228 is resistant to the antifungal drug FK506, but some of its progeny have lost this phenotype. We speculate that this may also generate progeny with increased fitness in response to other selective pressures not analyzed. Our study suggests that caution should be exercised when studying AD hybrids, and especially that the analysis of one single colony should be avoided.

Hybridization is an important mechanism for speciation in angiosperms by generating novel gene combinations and distinct phenotypes (Paun *et al.* 2009; Rieseberg *et al.* 2003; Soltis and Soltis 2009). In many cases, hybridization between different species or subspecies is prevented by prezygotic barriers, or the hybrid is sterile or exhibits reduced fertility because of genetic divergence between the parental strains. In some cases, however, the hybrid can also be stably fertile via either hybridization followed with genome doubling (allopolyploidy) or introgressive hybridization without a change in chromosome number (homoploidy) (Gompert *et al.* 2006; Rieseberg *et al.* 2003; Salazar *et al.* 2010). Then, the stable fertile hybrid can be reproductively isolated from its parental species by postzygotic barriers, and is therefore regarded as a novel species (Gutierrez-Marcos *et al.* 2003). In *C. neoformans*, there is no evidence that the AD hybrid has undergone genome doubling after hybridization between serotype A and D strains. Our FACS and CGH analyses found that the AD hybrid isolates have similar DNA content as the diploid, but not tetraploid, isolates. Thus, allopolyploid speciation does not likely occur in AD hybrids. Although introgression between serotype A and D genomes has been detected, it is on a smaller scale and occurred historically (Kavanaugh *et al.* 2006). The authors of a previous study identified an approximately 40-kb region (~0.2% of the genome) that is nearly identical (98.5%) between the serotype A and D genomes (Kavanaugh *et al.* 2006). This identity island may result from a nonreciprocal transfer event from serotype A to D approximately 2 MYA (Kavanaugh *et al.* 2006). This introgression event likely occurred during the hybridization process of serotype A and D strains and was facilitated by transposon-mediated DNA transfer. This is the only introgression region identified in *C. neoformans* thus far. No AD hybrid genomic sequence is currently available, mostly because of the difficulties of assembling the sequence reads into two genomes that share 85% to 90% sequence identity. Thus, the potential recombination events in the AD hybrid isolates are largely unknown. However, given the high percentage of transposons in the *C. neoformans* genome (Loftus *et al.* 2005), extensive introgression and recombination events may occur in AD hybrids during the long evolutionary period, which may be able to generate homoploidy to result in speciation. We observed that AD hybrid isolates have unique sequence types compared with their parental isolates, although they are highly related phylogenetically. This finding suggests limited gene flow between AD hybrids and the haploid population. Although the causes of genetic divergence in AD hybrid isolates are not known, this may be an early sign of speciation of the AD hybrid. In addition, most AD hybrid isolates cannot mate with haploid parental isolates in the laboratory, which may result in reproductive isolation and ultimately speciation of the AD hybrid.

We observed that most AD hybrid isolates are resistant to the antifungal drug FK506 (Figure 7). Together with the previous observation that AD hybrids are resistant to ultraviolet light (Lin *et al.* 2007), we hypothesize that the AD hybrid has hybrid vigor that may be attributable to the genome combination and genomic dynamics. Regulation of the genomic organization is a rapid and efficient route for human fungal pathogens to generate favorable phenotypes. For example, *C. neoformans* becomes resistant to fluconazole by chromosome duplication (Sionov *et al.* 2010), and aneuploidy and the formation of novel chromosomes contributes to antifungal drug resistance in *C. albicans* and *Candida glabrata* (Polakova *et al.* 2009; Selmecki *et al.* 2006; Sionov *et al.* 2010).

The AD hybrid genome is highly dynamic. We detected both partial and complete chromosome loss and duplication in some AD hybrid isolates (Figure 3). Progeny P5 of strain CDC228 has a distinct genomic organization resulting from the partial loss of the serotype A copy of Chrs 14 and 10 (corresponding to serotype D Chr 8 and Chr 10, respectively) and the complete loss of some other chromosomes (Figure 6). Partial chromosome loss may result in genome arrangement or the formation of novel chromosomes. Indeed, the loss of resistance to the antifungal drug FK506 in P5 may be attributable to this distinct genomic organization.

The AD hybrid generates population diversity efficiently. Although most progeny of AD hybrids are sterile and have low viability, the surviving progeny may have considerable hybrid vigor. Hybrid vigor may promote the global spread of AD hybrids, which may contribute to the preservation and exportation of the rare *MAT***a** genome. This is common in some plant species. The *Senecia* species are native in France, but the interspecific hybrid resulting from hybridization and allopolyploidy has spread to England (Abbott and Lowe 2004). *Tragopogon* is native to Euroasia, but the hybrid species invaded into North America (Soltis and Soltis 1999).

In summary, we characterized in this study the genetic diversity and genomic organization of the *C. neoformans* AD hybrid. Our results demonstrate that the AD hybrid originated from hybridization between serotype A and D strains and arose multiple times via independent hybridization events. The AD hybrid genome is highly dynamic, providing an efficient way for the AD hybrid to generate population dynamics and hybrid vigor. The hybrid vigor of the AD hybrid may facilitate it to spread globally, which may preserve the rare *MAT***a** allele. The potential contribution of the AD hybrid to speciation is an interesting topic for future investigation.

## Supplementary Material

Supporting Information
