# Supplementary material for: Genetic Diversity and Genomic Plasticity of Cryptococcus neoformans AD Hybrid Strains
Source: G3 (Bethesda). 2012 Jan 1;2(1):83–97. doi: 10.1534/g3.111.001255 (PMC3276195; doi:10.1534/g3.111.001255)
Supplement: Supporting Information [file supp_2_1_83__index.html]

Supporting Information 

# Genetic Diversity and Genomic Plasticity of *Cryptococcus neoformans* AD Hybrid Strains

## Supporting Information for Li *et al.*, 2012

**Files in this Data Supplement:**

- Supporting Information - Figures S1-S4 and Table S1 (PDF, 1.8 MB)
- Figure S1 - Whole genome comparison between serotype A strain H99 and serotype D strain JEC21 using BLASTn and ATC tool (PDF, 888 KB)
- Figure S2 - Molecular determination of the serotype/mating type of the C. neoformans isolates based on PCR amplification of the STE20 genes (PDF, 376 KB)
- Figure S3 - Phylogenetic organization of serotype D isolates based on *IGS*, *URE1*, *GPD1*, *LAC1*, and *MPD1* (PDF, 472 KB)
- Figure S4 - Informative paired allele graphs of three MLST markers (*GPD1*, *LAC1*, and *MPD1*) (PDF, 52 KB)
- Table S1 - The sequence types (STs) of *C. neoformans* isolates analyzed in this study (PDF, 92 KB)
