# Supplementary material for: Genetic Diversity and Genomic Plasticity of Cryptococcus neoformans AD Hybrid Strains
Source: G3 (Bethesda). 2012 Jan 1;2(1):83–97. doi: 10.1534/g3.111.001255 (PMC3276195; doi:10.1534/g3.111.001255)
Supplement: Supporting Information [file supp_2.1.83_001255SI.pdf]

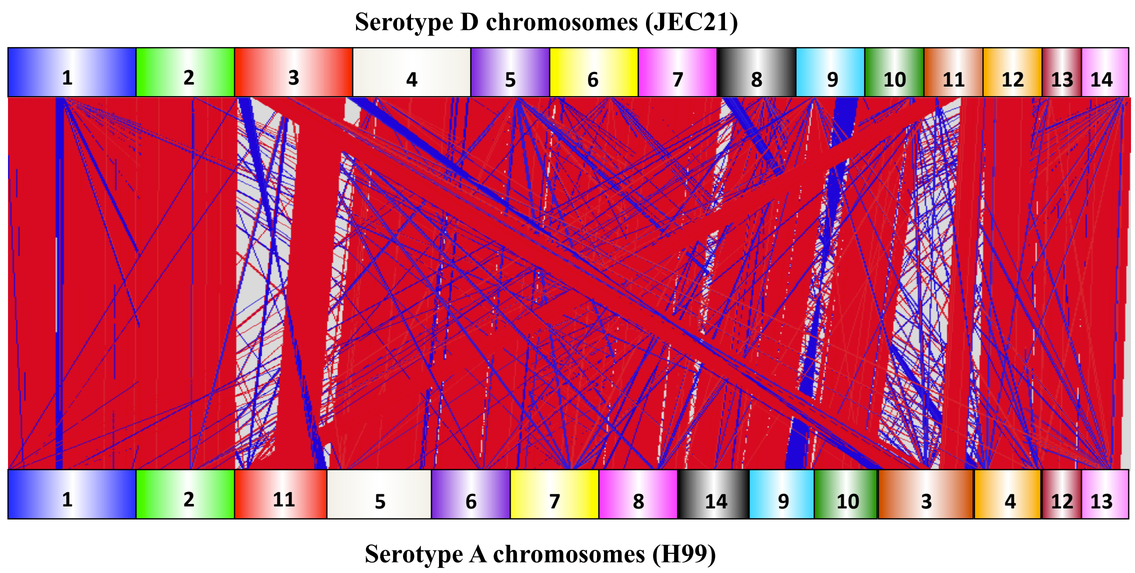

Figure S1 Whole genome comparison between serotype A strain H99 and serotype D strain JEC21 using BLASTn and ATC tool. Red and blue blocks (lines) represent forward and reverse matches of the DNA sequence between strains JEC21 and H99, respectively.

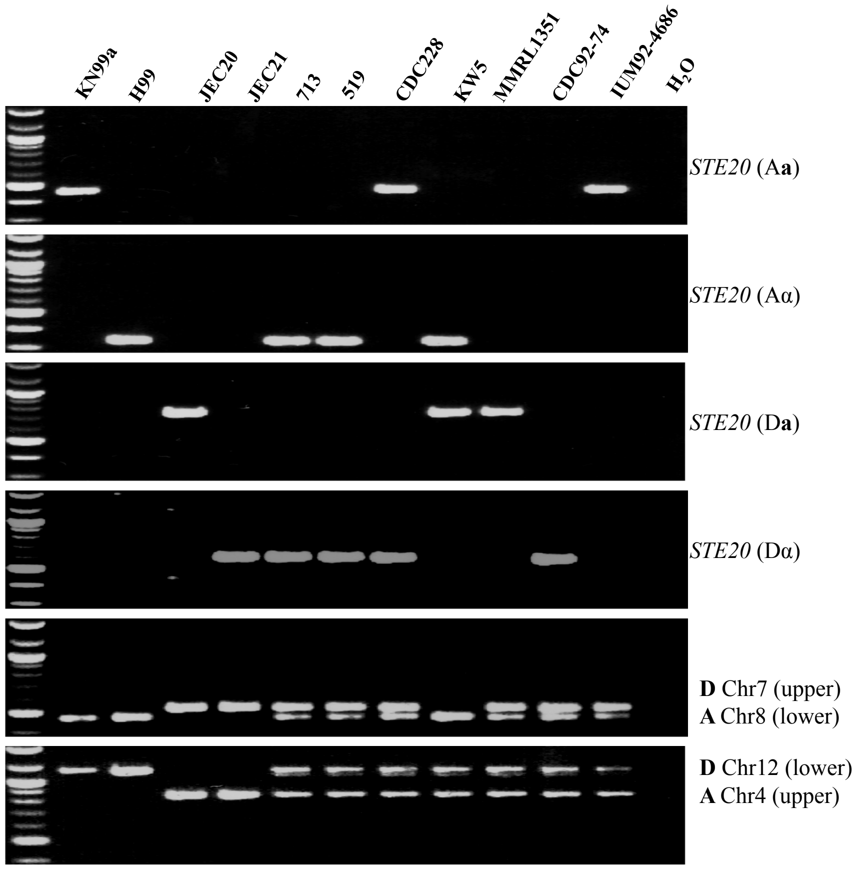

Figure S2 Molecular determination of the serotype/mating type of the *C. neoformans* isolates based on PCR amplification of the *STE20* genes.

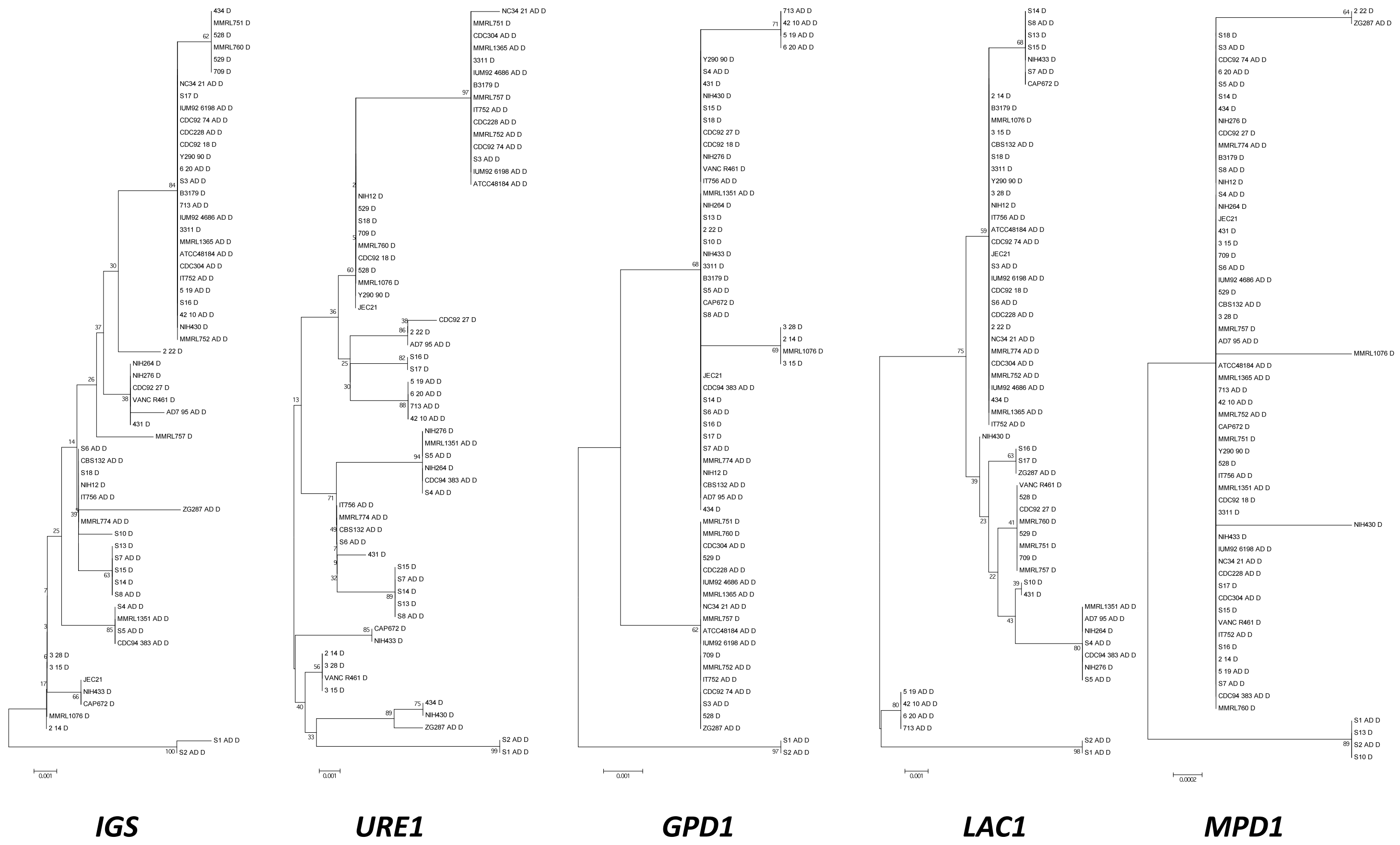

Figure S3 Phylogenetic organization of serotype D isolates based on *IGS*, *URE1*, *GPD1*, *LAC1*, and *MPD1*.

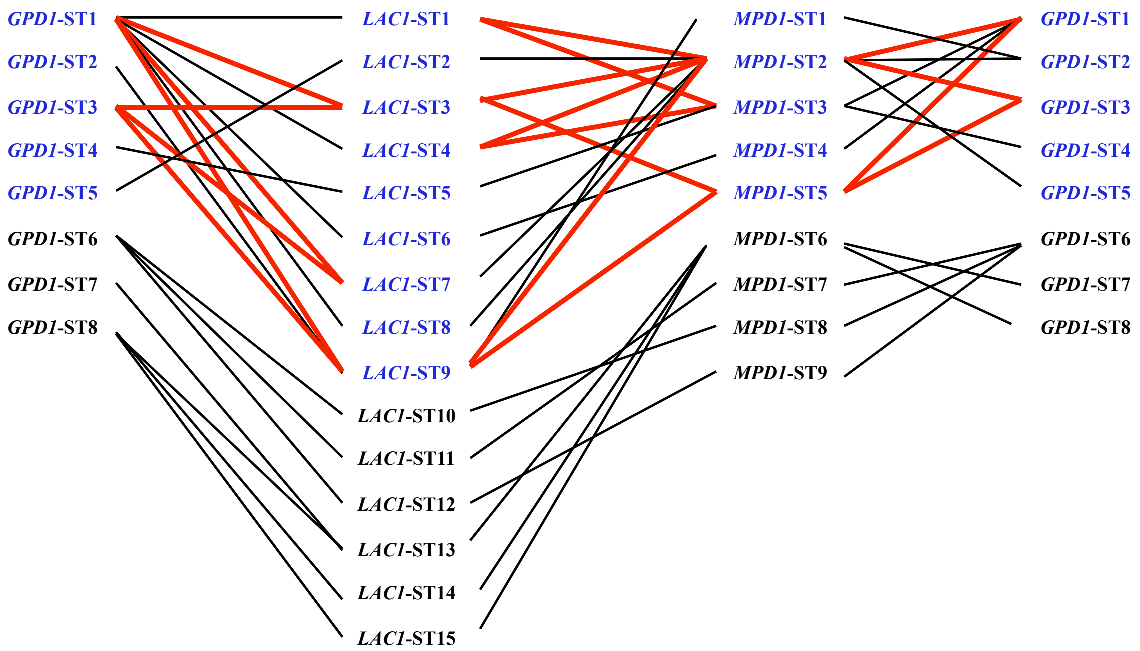

Figure S4 Informative paired allele graphs of three MLST markers (*GPD1*, *LAC1*, and *MPD1*). An hourglass shape (shown in red) indicates the presence of all four possible pairs of alleles and serves as evidence for recombination. Serotype A MLST STs are shown in black and serotype D STs are in blue.

Table S1 The sequence types (STs) of *C. neoformans* isolates analyzed in this study.

Strain	Serotype	Genome analyzed	*IGS*	*URE1*	*GPD1*	*LAC1*	*MPD1*	ST (5 markers)
AD6-93	AD	D	5	14	4	5	3	15
AD7-97	AD	D	11	14	4	5	3	27
AD6-38	AD	D	15	7	3	9	2	16
AD7-91	AD	D	10	8	1	8	2	20
AD7-85	AD	D	10	8	1	8	2	20
AD7-69	AD	D	17	11	1	9	2	12
AD7-75	AD	D	16	4	1	1	2	24
AD2-71	AD	D	16	4	1	1	2	24
AD7-95	AD	D	18	6	1	8	2	4
713	AD	D	15	17	5	2	2	1
42-10	AD	D	15	17	5	2	2	1
519	AD	D	15	17	5	2	2	1
620	AD	D	15	17	5	2	2	1
ATCC48184	AD	D	6	7	3	9	2	7
CDC228	AD	D	15	7	3	9	2	16
CDC304	AD	D	15	7	3	9	2	16
CDC92-74	AD	D	15	7	3	9	2	16
CDC94-383	AD	D	10	8	1	8	2	20
CBS132	AD	D	17	11	1	9	2	12
IT752	AD	D	15	7	3	9	2	16
IT756	AD	D	17	11	1	9	2	12
IUM92-4686	AD	D	15	7	3	9	2	16
IUM92-6198	AD	D	15	7	3	9	2	16
MMRL752	AD	D	15	7	3	9	2	16
MMRL774	AD	D	17	11	1	9	2	12
MMRL1351	AD	D	10	8	1	8	2	20
MMRL1365	AD	D	15	7	3	9	2	16
NC34-21	AD	D	15	13	3	9	2	28
ZG287	AD	D	12	1	3	3	5	6
AD7-71	D	D	13	5	1	4	3	10
AD2-62	D	D	16	4	1	1	3	21
AD2-95	D	D	16	4	1	1	2	24
AD2-96	D	D	16	4	1	1	2	24
AD3-14	D	D	4	10	1	3	2	23
AD3-13	D	D	4	10	1	3	2	23
AD8-62	D	D	17	9	1	9	2	8
2-14	D	D	1	3	2	9	2	14
2-22	D	D	14	6	1	9	5	9
3-15	D	D	1	3	2	9	2	14

3-28	D	D	1	3	2	9	2	14
431	D	D	3	2	1	4	2	22
434	D	D	2	15	1	9	2	17
528	D	D	2	9	3	7	2	25
529	D	D	2	9	3	7	2	25
709	D	D	2	9	3	7	2	25
3311	D	D	4	7	1	9	2	31
B3179	D	D	4	7	1	9	2	31
CAP672	D	D	9	12	1	1	2	5
CDC92	D	D	4	9	1	9	2	18
CDC92	D	D	3	16	1	7	2	11
MMRL751	D	D	2	7	3	7	2	30
MMRL757	D	D	8	7	3	7	2	29
MMRL760	D	D	2	9	3	7	2	25
MMRL1076	D	D	1	9	2	9	1	13
NIH12	D	D	17	9	1	9	2	8
VANC.R461	D	D	3	3	1	7	2	19
Y290-90	D	D	4	9	1	9	2	18
JEC21	D	D	9	9	1	9	2	26
NIH264	D	D	7	8	1	8	2	3
NIH276	D	D	7	8	1	8	2	3
NIH430	D	D	15	15	1	6	4	2
NIH433	D	D	9	12	1	1	2	5
AD6-93	AD	A	22	18	6	11	7	35
AD7-97	AD	A	22	18	6	11	7	35
AD6-38	AD	A	21	19	6	10	8	38
AD7-91	AD	A	20	20	8	13	6	34
AD7-85	AD	A	20	20	8	13	6	34
AD7-69	AD	A	22	20	7	13	6	37
AD7-75	AD	A	22	20	8	15	6	39
AD2-71	AD	A	22	20	8	15	6	39
AD7-95	AD	A	19	20	8	13	6	36
713	AD	A	22	20	8	14	6	32
42-10	AD	A	22	20	8	14	6	32
5-19	AD	A	22	20	8	14	6	32
6-20	AD	A	22	20	8	14	6	32
CDC228	AD	A	21	19	6	12	9	33
CDC304	AD	A	21	19	6	12	9	33
CDC92-74	AD	A	21	19	6	12	9	33
IT752	AD	A	21	19	6	12	9	33
KW5	AD	A	20	20	8	13	6	34
MMRL752	AD	A	21	19	6	12	9	33

MMRL774	AD	A	22	20	7	13	6	37
NC34-21	AD	A	21	19	6	12	9	33
ZG287	AD	A	22	20	7	13	6	37
ZG290	AD	A	21	19	6	12	9	33
