# Supplementary material for: Genetic Diversity and Genomic Plasticity of Cryptococcus neoformans AD Hybrid Strains
Source: G3 (Bethesda). 2012 Jan 1;2(1):83–97. doi: 10.1534/g3.111.001255 (PMC3276195; doi:10.1534/g3.111.001255)
Supplement: Supporting Information [file supp_2.1.83_FigureS1.pdf]

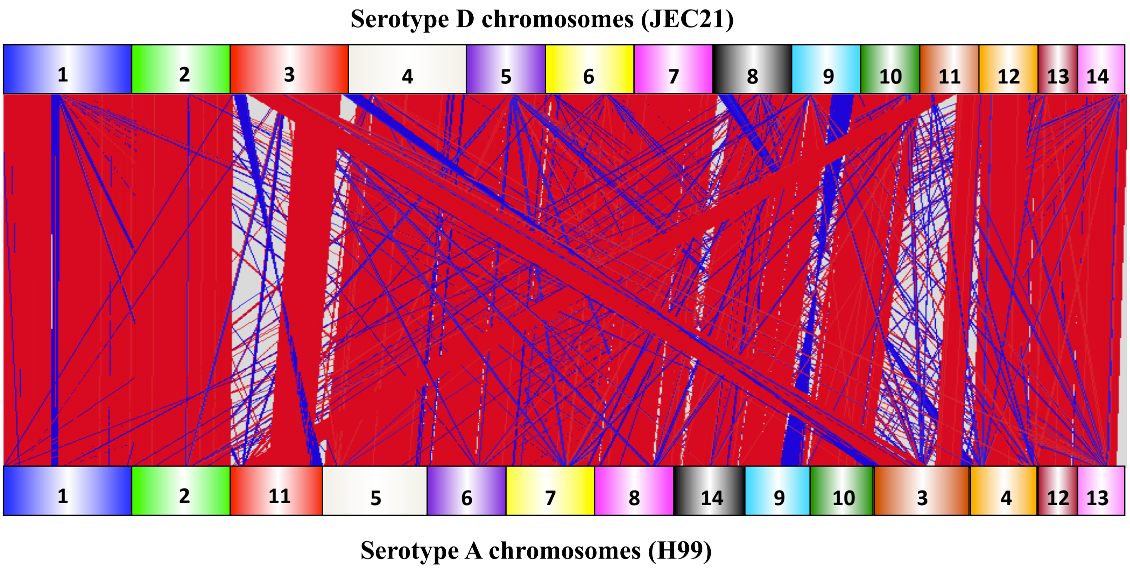

Figure S1 Whole genome comparison between serotype A strain H99 and serotype D strain JEC21 using BLASTn and ATC tool. Red and blue blocks (lines) represent forward and reverse matches of the DNA sequence between strains JEC21 and H99, respectively.
