# Supplementary material for: Genetic Diversity and Genomic Plasticity of Cryptococcus neoformans AD Hybrid Strains
Source: G3 (Bethesda). 2012 Jan 1;2(1):83–97. doi: 10.1534/g3.111.001255 (PMC3276195; doi:10.1534/g3.111.001255)
Supplement: Supporting Information [file supp_2.1.83_FigureS2.pdf]

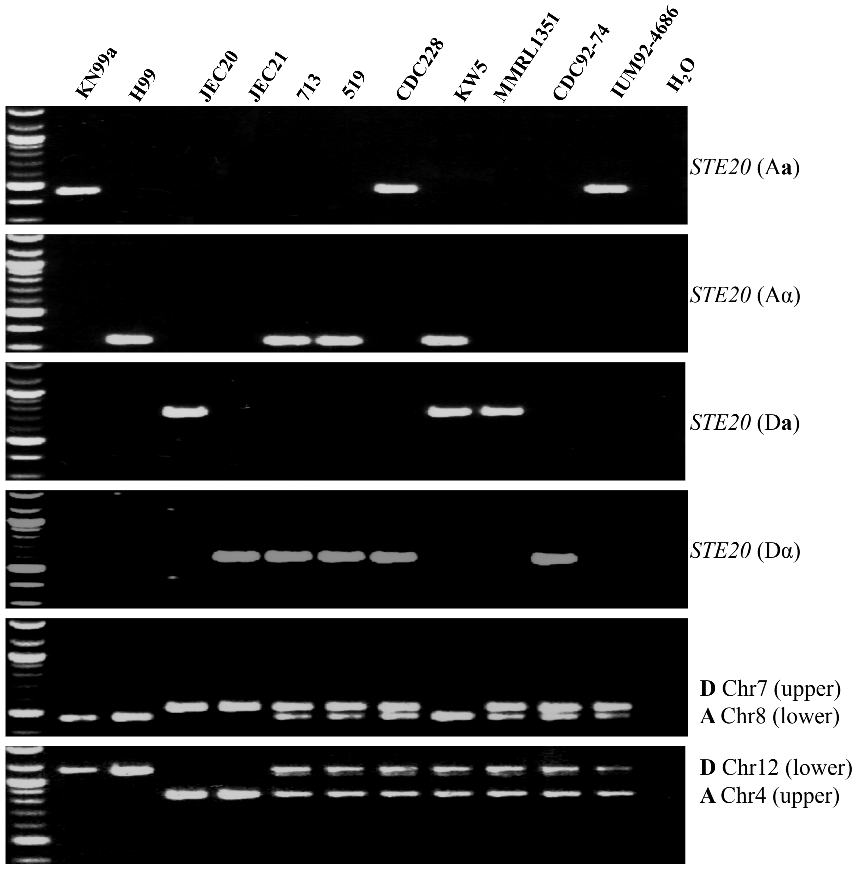

Figure S2 Molecular determination of the serotype/mating type of the *C. neoformans* isolates based on PCR amplification of the *STE20* genes.
