# Supplementary figures and images for: Genetic Diversity and Genomic Plasticity of Cryptococcus neoformans AD Hybrid Strains
Source: G3 (Bethesda). 2012 Jan 1;2(1):83–97. doi: 10.1534/g3.111.001255 (PMC3276195; doi:10.1534/g3.111.001255)

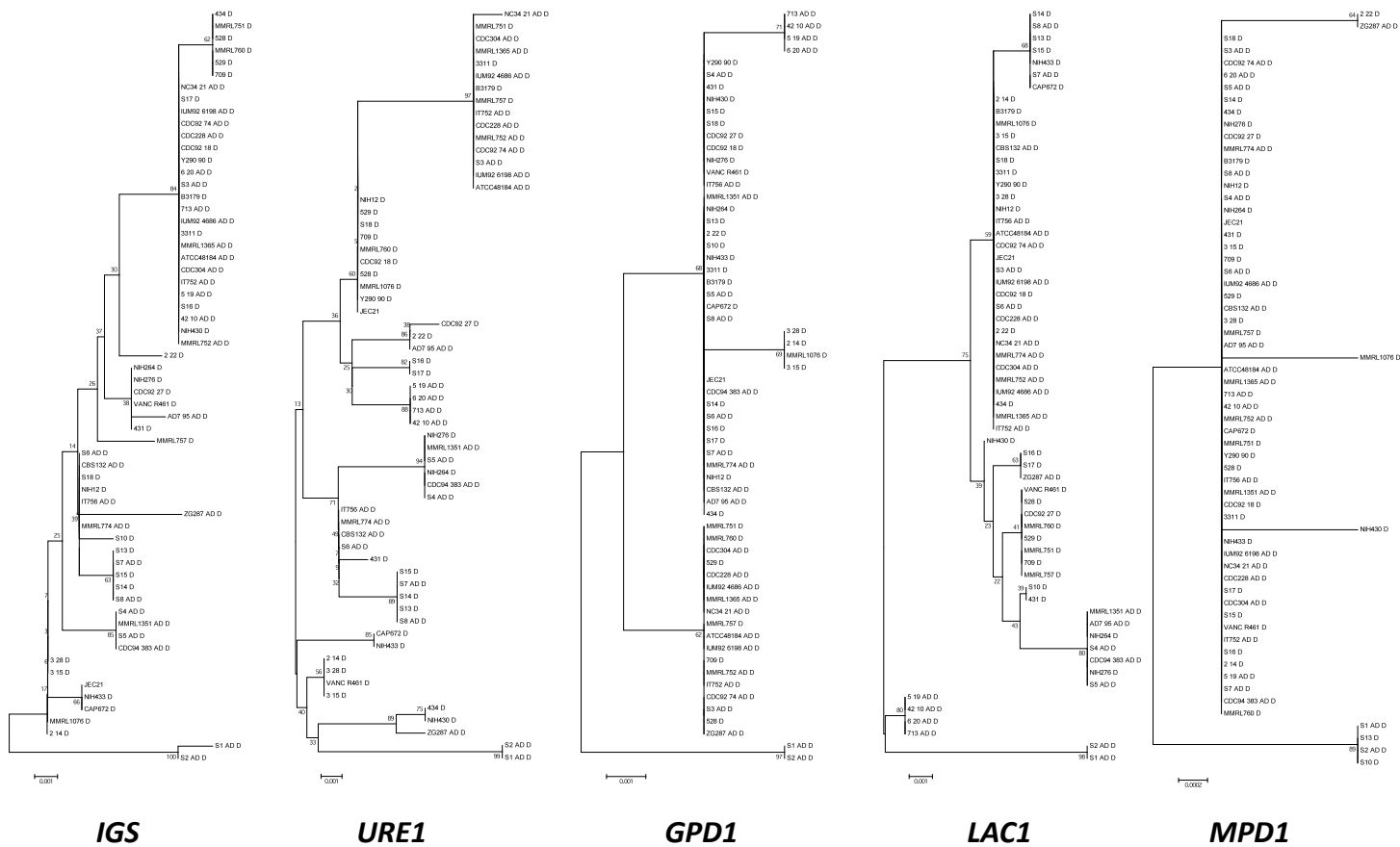

**Figure S3** Phylogenetic organization of serotype D isolates based on *IGS*, *URE1*, *GPD1*, *LAC1*, and *MPD1*.

Supplement: Supporting Information [file supp_2.1.83_FigureS3.pdf]
