# Supplementary material for: Genetic Diversity and Genomic Plasticity of Cryptococcus neoformans AD Hybrid Strains
Source: G3 (Bethesda). 2012 Jan 1;2(1):83–97. doi: 10.1534/g3.111.001255 (PMC3276195; doi:10.1534/g3.111.001255)
Supplement: Supporting Information [file supp_2.1.83_FigureS4.pdf]

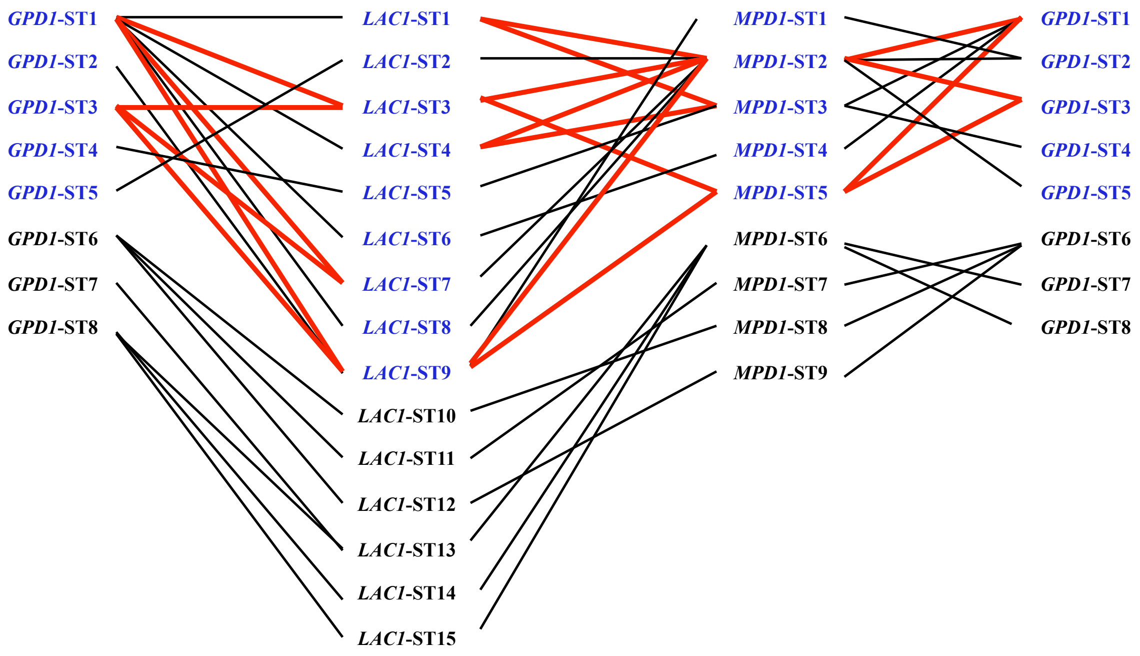

Figure S4 Informative paired allele graphs of three MLST markers (*GPD1*, *LAC1*, and *MPD1*). An hourglass shape (shown in red) indicates the presence of all four possible pairs of alleles and serves as evidence for recombination. Serotype A MLST STs are shown in black and serotype D STs are in blue.
