# Supplementary material for: Genetic Diversity and Genomic Plasticity of Cryptococcus neoformans AD Hybrid Strains
Source: G3 (Bethesda). 2012 Jan 1;2(1):83–97. doi: 10.1534/g3.111.001255 (PMC3276195; doi:10.1534/g3.111.001255)
Supplement: Supporting Information [file supp_2.1.83_TableS1.pdf]

Table S1 The sequence types (STs) of *C. neoformans* isolates analyzed in this study.

Strain	Serotype	Genome analyzed	*IGS*	*URE1*	*GPD1*	*LAC1*	*MPD1*	ST (5 markers)
AD6-93	AD	D	5	14	4	5	3	15
AD7-97	AD	D	11	14	4	5	3	27
AD6-38	AD	D	15	7	3	9	2	16
AD7-91	AD	D	10	8	1	8	2	20
AD7-85	AD	D	10	8	1	8	2	20
AD7-69	AD	D	17	11	1	9	2	12
AD7-75	AD	D	16	4	1	1	2	24
AD2-71	AD	D	16	4	1	1	2	24
AD7-95	AD	D	18	6	1	8	2	4
713	AD	D	15	17	5	2	2	1
42-10	AD	D	15	17	5	2	2	1
519	AD	D	15	17	5	2	2	1
620	AD	D	15	17	5	2	2	1
ATCC48184	AD	D	6	7	3	9	2	7
CDC228	AD	D	15	7	3	9	2	16
CDC304	AD	D	15	7	3	9	2	16
CDC92-74	AD	D	15	7	3	9	2	16
CDC94-383	AD	D	10	8	1	8	2	20
CBS132	AD	D	17	11	1	9	2	12
IT752	AD	D	15	7	3	9	2	16
IT756	AD	D	17	11	1	9	2	12
IUM92-4686	AD	D	15	7	3	9	2	16
IUM92-6198	AD	D	15	7	3	9	2	16
MMRL752	AD	D	15	7	3	9	2	16
MMRL774	AD	D	17	11	1	9	2	12
MMRL1351	AD	D	10	8	1	8	2	20
MMRL1365	AD	D	15	7	3	9	2	16
NC34-21	AD	D	15	13	3	9	2	28
ZG287	AD	D	12	1	3	3	5	6
AD7-71	D	D	13	5	1	4	3	10
AD2-62	D	D	16	4	1	1	3	21
AD2-95	D	D	16	4	1	1	2	24
AD2-96	D	D	16	4	1	1	2	24
AD3-14	D	D	4	10	1	3	2	23
AD3-13	D	D	4	10	1	3	2	23
AD8-62	D	D	17	9	1	9	2	8
2-14	D	D	1	3	2	9	2	14
2-22	D	D	14	6	1	9	5	9
3-15	D	D	1	3	2	9	2	14

3-28	D	D	1	3	2	9	2	14
431	D	D	3	2	1	4	2	22
434	D	D	2	15	1	9	2	17
528	D	D	2	9	3	7	2	25
529	D	D	2	9	3	7	2	25
709	D	D	2	9	3	7	2	25
3311	D	D	4	7	1	9	2	31
B3179	D	D	4	7	1	9	2	31
CAP672	D	D	9	12	1	1	2	5
CDC92	D	D	4	9	1	9	2	18
CDC92	D	D	3	16	1	7	2	11
MMRL751	D	D	2	7	3	7	2	30
MMRL757	D	D	8	7	3	7	2	29
MMRL760	D	D	2	9	3	7	2	25
MMRL1076	D	D	1	9	2	9	1	13
NIH12	D	D	17	9	1	9	2	8
VANC.R461	D	D	3	3	1	7	2	19
Y290-90	D	D	4	9	1	9	2	18
JEC21	D	D	9	9	1	9	2	26
NIH264	D	D	7	8	1	8	2	3
NIH276	D	D	7	8	1	8	2	3
NIH430	D	D	15	15	1	6	4	2
NIH433	D	D	9	12	1	1	2	5
AD6-93	AD	A	22	18	6	11	7	35
AD7-97	AD	A	22	18	6	11	7	35
AD6-38	AD	A	21	19	6	10	8	38
AD7-91	AD	A	20	20	8	13	6	34
AD7-85	AD	A	20	20	8	13	6	34
AD7-69	AD	A	22	20	7	13	6	37
AD7-75	AD	A	22	20	8	15	6	39
AD2-71	AD	A	22	20	8	15	6	39
AD7-95	AD	A	19	20	8	13	6	36
713	AD	A	22	20	8	14	6	32
42-10	AD	A	22	20	8	14	6	32
5-19	AD	A	22	20	8	14	6	32
6-20	AD	A	22	20	8	14	6	32
CDC228	AD	A	21	19	6	12	9	33
CDC304	AD	A	21	19	6	12	9	33
CDC92-74	AD	A	21	19	6	12	9	33
IT752	AD	A	21	19	6	12	9	33
KW5	AD	A	20	20	8	13	6	34
MMRL752	AD	A	21	19	6	12	9	33

MMRL774	AD	A	22	20	7	13	6	37
NC34-21	AD	A	21	19	6	12	9	33
ZG287	AD	A	22	20	7	13	6	37
ZG290	AD	A	21	19	6	12	9	33
